# Annulation of 1*H*-pyrrole-2,3-diones by thioacetamide: an approach to 5-azaisatins

**DOI:** 10.3762/bjoc.15.32

**Published:** 2019-02-07

**Authors:** Aleksandr I Kobelev, Ekaterina E Stepanova, Maksim V Dmitriev, Andrey N Maslivets

**Affiliations:** 1 Department of Chemistry, Perm State University, ul. Bukireva 15, Perm 614990, Russian Federation

**Keywords:** annulation, domino reactions, isatin, nitrogen heterocycles, thioamides

## Abstract

A novel approach to 1*H*-pyrrolo[3,2-*c*]pyridine-2,3-diones (5-azaisatins) has been developed via an unprecedented annulation of pyrrolo[2,1-*c*][1,4]benzoxazine-1,2,4-triones by thioacetamide. A new way of C–H functionalization of thioacetamide has been discovered. The reaction proceeds under green catalyst-free conditions.

## Introduction

Omnipresent among natural compounds and drugs, the indole core is a privileged scaffold for library design and drug discovery [1]. Its dioxo derivative, isatin (1*H*-indole-2,3-dione), is an important building block for the construction of diverse indole-based compounds which are in great request in medicinal chemistry as they exhibit a variety of biological activities [2–9].

**Scheme 1:**
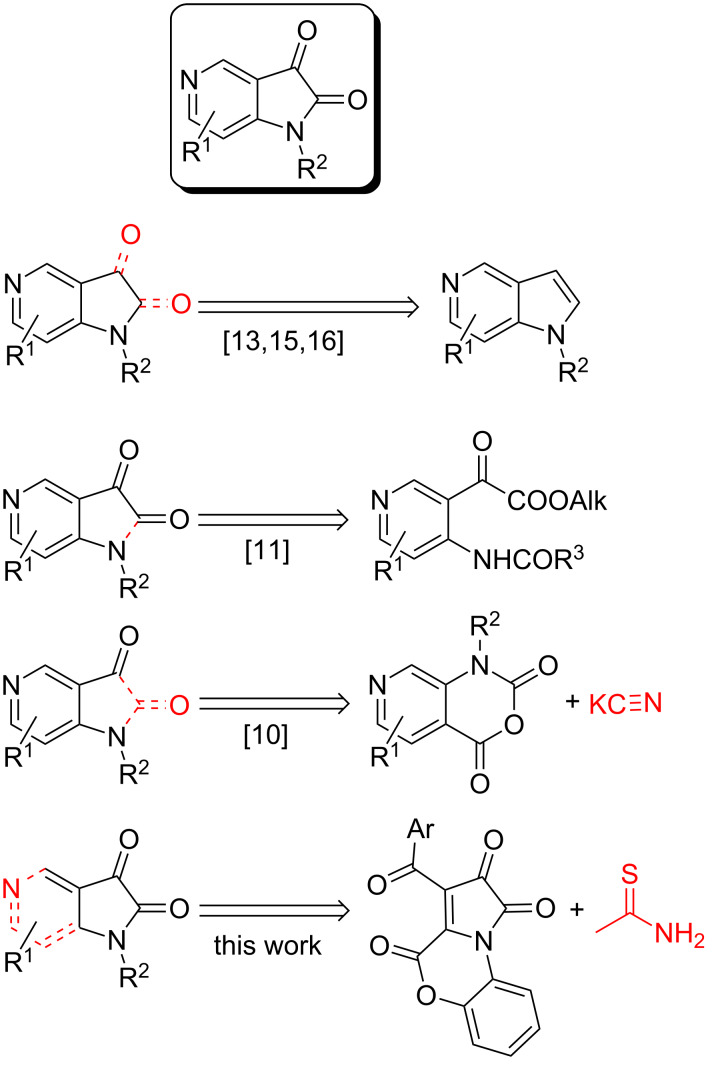
Approaches to the synthesis of the 5-azaisatin core.

5-Azaisatins (1*H*-pyrrolo[3,2-*c*]pyridine-2,3-diones) are a useful but sparingly studied kind of isatin’s aza isosteres [10–16]. Presently, few synthetic approaches towards this tempting scaffold are reported in the literature. They can be divided into two groups: oxidation of pyrrolo[3,2-*c*]pyridines [13,15–16] and annulation of pyridine by a pyrrole-2,3-dione moiety (cyclization of 2-(4-((alkoxycarbonyl)amino)pyridin-3-yl)-2-oxoacetates [11], interaction of 5-azaisatoic anhydride with cyanides followed by hydrolysis [10], Scheme 1).

Herein, we report a distinct synthetic approach to 5-azaisatins via annulation of pyrrole-2,3-dione by a pyridine ring based on an unprecedented interaction of 1*H*-pyrrole-2,3-diones with thioacetamide (Scheme 1).

## Results and Discussion

**Scheme 2:**
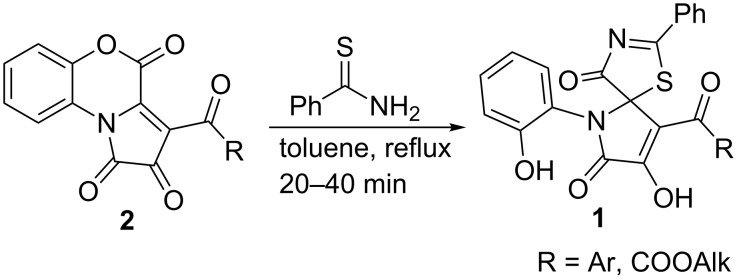
Our previous work on the interaction of PBTs **2** with thioamides.

Previously, we reported an efficient catalyst-free synthesis of spiro[thiazolo-5,2'-pyrroles] **1** via interaction of pyrrolobenzoxazinetriones (PBTs) **2** with thiobenzamide (Scheme 2) [17–18].

**Scheme 3:**
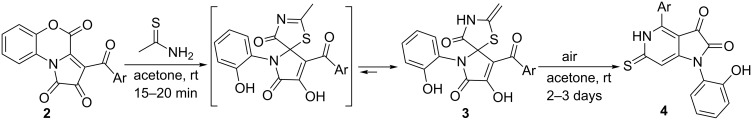
Interaction of PBTs **2** with thioacetamide.

In continuation of this research [17–18], and in order to extend the scope of the reaction, we attempted to involve the simplest aliphatic thioamide, thioacetamide, in the interaction with PBTs **2**. As a result, we obtained the expected spiro[thiazolo-5,2'-pyrroles] **3**, which were found to exist in a form with an *exo*-methylene group according to the NMR spectra (Scheme 3) [19]. Unfortunately, the products **3** were found to be labile and hard to isolate and purify. During our attempts to obtain pure compounds **3**, we observed that their yellow acetone solutions became orange when stored for several hours in contact with the atmosphere, and after several days of storage, they had formed orange precipitates. The precipitates were isolated and analyzed. We established that they were formed by pyrrolo[3,2-*c*]pyridine-2,3-diones **4** (Scheme 3). The structure of compounds **4** was unequivocally confirmed by a single crystal X-ray analysis (CCDC 1877232).

**Scheme 4:**
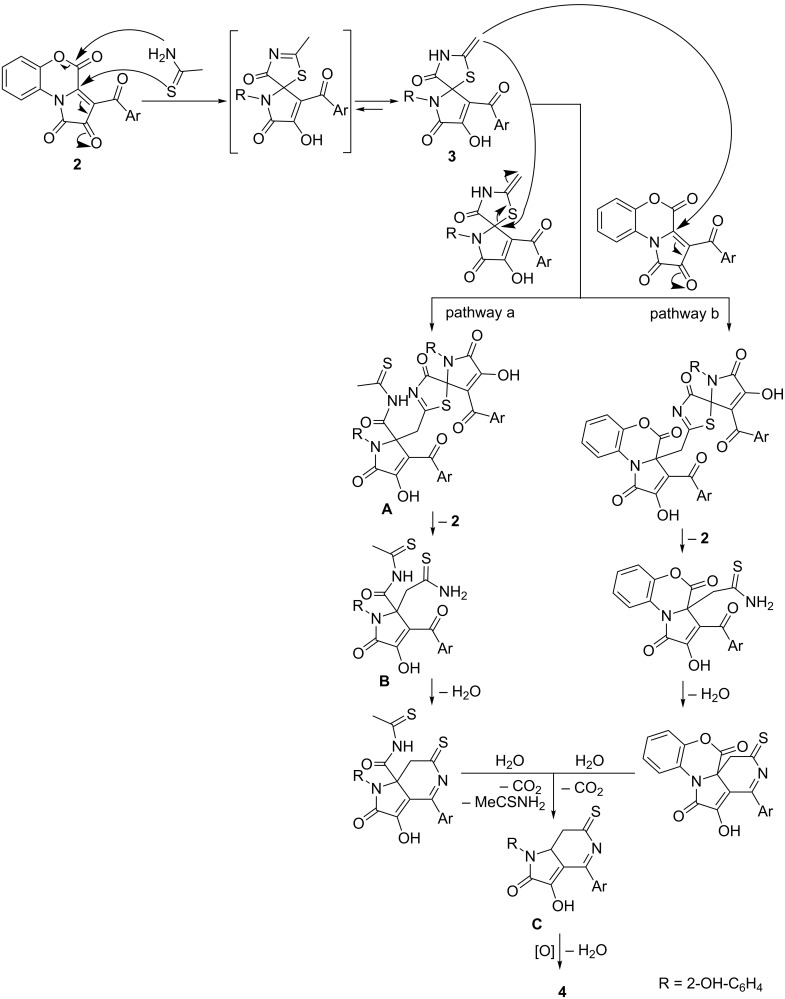
Plausible pathways for the formation of compound **4**.

We assume that the formation of compounds **4** occurred through a cascade of several reactions (Scheme 4, pathway a). The first step was the addition of thioacetamide to PBT **2** by subsequent nucleophilic attacks of S and N atoms. The formed compound **3** had a strong nucleophilic center – the *exo*-methylene group, which could attack the other molecule of compound **3** to substitute the S atom and form adduct **A**. Adduct **A** underwent a decomposition by releasing a molecule of compound **2** [18]. The formed compound **B** underwent intramolecular cyclization followed by hydrolysis of the imide group with subsequent decarboxylation and formation of the intermediate **C** that was oxidized by the air oxygen to form compound **4**. Possibly, the formation of compound **4** proceeded through an alternative pathway (Scheme 4, pathway b). In this case, the formed compound **3** reacted with a molecule of the PBT **2** resulting in an intermediate that underwent a similar pathway to afford compound **4**.

To confirm the proposed pathway and optimize the reaction conditions, we carried out a series of experiments using the PBT **2a**.

First, the solvent effect was checked (Table 1). We found that the solvent played a crucial role, and the best yields were observed in hygroscopic polar solvents (Table 1, entries 1, 4, 7, 8, 11) and 1,4-dioxane (Table 1, entry 9). The temperature effect was also checked (Table 1, entries 1–3), and it was found to be insignificant.

**Table 1:** Optimization of the reaction conditions^a^.

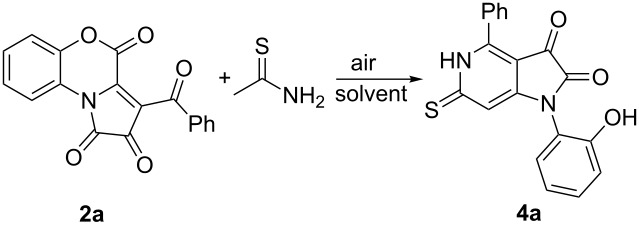			

entry	solvent	temp. (°C)	yield^b^

1	acetone	rt	21
2	acetone	56	18
3	acetone	–16	17
4	acetonitrile	rt	23
5	chloroform	rt	6
6	DCM	rt	traces
7	DMF	rt	28
8	DMSO	rt	28
9	1,4-dioxane	rt	20
10	ethyl acetate	rt	12
11	NMP	rt	22
12	toluene	rt	5
13	acetone^c^	rt	7

^a^Typical procedure: A solution of PBT **2a** (10 mg, 0.031 mmol) and thioacetamide (2.3 mg, 0.031 mmol) in 0.5 mL of a solvent was kept under air atmosphere for 2 days. ^b^HPLC yields. ^c^Five-fold excess of thioacetamide was used.

Having noticed the influence of the solvent hygroscopicity, we examined the influence of water added to the reaction mixture. The experiment was carried out in acetone, DMSO, and NMP under the typical procedure conditions from Table 1 (entries 1, 8, 11); different amounts of water (10, 20, 40, 80 and 100 µL) were added to the reaction mixtures, and the yields of the desired compound **4a** were determined by HPLC. As a result, we found that the addition of water did not affect the yield of compound **4a** or even slightly lowered it.

Having determined the optimal conditions of the reactions, we turned our attention to the main distinction of the proposed pathways of formation of compounds **4** – the particle that interacted with the spiro[thiazolo-5,2'-pyrroles] **3**. It could be either a molecule of the spiro[thiazolo-5,2'-pyrrole] **3** or a molecule of the PBT **2**. To determine the most possible one, we investigated the effect of the ratio of the reagents. When we used excessive amounts of thioacetamide (fivefold excess to PBT **2a**), a significant decrease in the yield of **4a** was observed (in acetone the yield was 7% counting on PBT **2a**). When an excessive amount of PBT **2a** (double excess to thioacetamide) was used, a notable increase in yield of **4a** was observed (the yield was 56% in acetone and 60% in DMSO counting on thioacetamide). These can indicate that the pathway through a PBT (Scheme 4, pathway b) is more presumable.

**Scheme 5:**
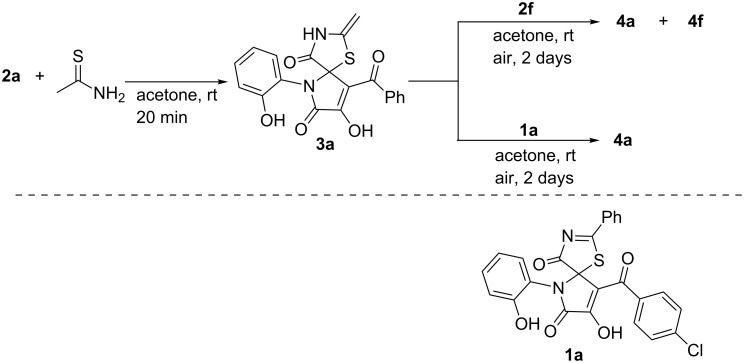
Experiments on the intermolecular trapping of spiro[thiazolo-5,2'-pyrrole] **3a**.

In addition, we carried out two more experiments in order to determine the most presumable pathway of formation of compounds **4** (Scheme 4). PBT **2a** and thioacetamide (in ratio of 1:1) were kept in acetone for 20 min to afford a solution of compound **3a**. To the solution obtained 1 equivalent of either PBT **2f** or spiro[thiazolo-5,2'-pyrrole] **1a** [18] was added (Scheme 5). When **2a** was added, we observed the formation of a mixture of compounds **4a** and **4f** in a ratio of about 1:1, and in the case of addition of **1a**, no cross-product **4f** was observed (UPLC–MS). These can also confirm the pathway through a PBT **2** (Scheme 4, pathway b) to be more presumable. However, the absence of the cross-product **4f** can be also attributable to the steric hindrance created by the phenyl substituent in **1a** which violated the attack of the methylene group of **3a** on compound **1a**.

**Scheme 6:**
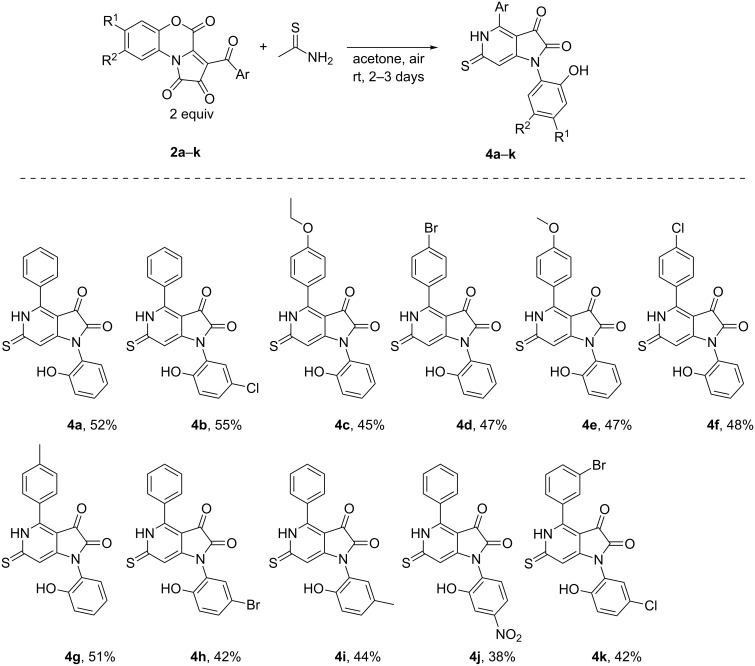
Exploration of the substrate scope.

Finally, we checked the substrate scope of the reaction. We found that the reaction proceeded well with various PBTs **2** (Scheme 6).

However, our attempts to change thioacetamide for cyanothioacetamide did not give any desirable products, as corresponding spiro[thiazolo-5,2'-pyrrole] **3** was formed very slowly and readily underwent multiple side-reactions. The involvement of propanethioamide and 2-phenylethanethioamide did not give any desirable results, too. These interactions provided mixtures of three isomeric adducts possibly corresponding to two stereoisomeric adducts (*cis* and *trans*) and an extra adduct of an unidentified side-reaction (UPLC–MS data).

**Scheme 7:**
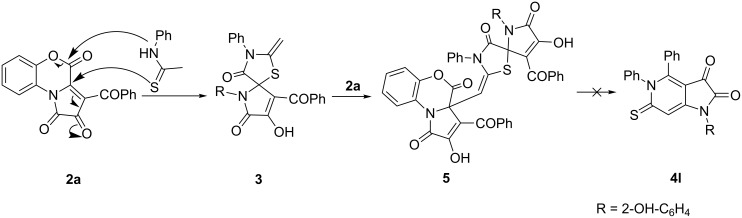
Interaction of PBT **2a** with *N*-phenylthioacetamide.

The interaction of PBT **2a** with *N*-phenylthioacetamide gave very interesting results. We found that the reaction of PBT **2a** and *N*-phenylthioacetamide (in ratios of both 2:1 and 1:1) carried out in acetone resulted in the formation of compound **5** (Scheme 7). The structure of compound **5** was proved by X-ray analysis (CCDC 1879686). Obviously, compound **5** corresponds to an intermediate proposed in pathway b (Scheme 4) which is one more evidence to support this pathway. Unfortunately, we did not succeed to transform compound **5** in the corresponding 1*H*-pyrrolo[3,2-*c*]pyridine-2,3-dione **4l**. The most possible explanation of this fact is the steric hindrance introduced by the *N*-phenyl substituent, which obstructs the thiazole ring opening.

## Conclusion

In summary, we have developed a novel approach to 1*H*-pyrrolo[3,2-*c*]pyridine-2,3-diones (5-azaisatins) via unprecedented annulation of pyrrolo[2,1-*c*][1,4]benzoxazine-1,2,4-triones by thioacetamide. The reaction proceeds under green catalyst-free conditions, at room temperature and air atmosphere. The products can be easily isolated directly from the reaction mixture without use of column chromatography. Moreover, the resulting compounds may be of pharmaceutical interest and can be starting materials for the synthesis of various aza analogs of isatin-based heterocycles. It also should be mentioned that we have observed an interesting thioacetamide-based reactive intermediate that can possibly serve for a catalyst-free C–H functionalization of thioacetamide and mild introduction of this fragment in other molecules, which is valuable in terms of synthetic organic chemistry.

## Supporting Information

File 1 Further experimental details, copies of ^1^H and ^13^C NMR spectra and X-ray crystal structure details.
