# Supplementary material for: Annulation of 1H-pyrrole-2,3-diones by thioacetamide: an approach to 5-azaisatins
Source: Beilstein J Org Chem. 2019 Feb 7;15:364–70. doi: 10.3762/bjoc.15.32 (PMC6369991; doi:10.3762/bjoc.15.32)
Supplement: File 1 — Further experimental details, copies of 1H and 13C NMR spectra and X-ray crystal structure details. [file Beilstein_J_Org_Chem-15-364-s001.pdf]

BEILSTEIN JOURNAL OF ORGANIC CHEMISTRY

Supporting Information

for

Annulation of 1*H*-pyrrole-2,3-diones by thioacetamide: an approach to 5-azaisatins

Aleksandr I. Kobelev, Ekaterina E. Stepanova, Maksim V. Dmitriev
and Andrey N. Maslivets

Beilstein J. Org. Chem. **2019,** *15,* 364–370. doi:10.3762/bjoc.15.32

Further experimental details, copies of ^{1}H and ^{13}C NMR spectra and X-ray crystal structure details

License and Terms: This is a supporting information file under the terms of the Creative Commons Attribution License (http://creativecommons.org/licenses/by/4.0). Please note that the reuse, redistribution and reproduction in particular requires that the authors and source are credited.
The license is subject to the *Beilstein Journal of Organic Chemistry* terms and conditions: (https://www.beilstein-journals.org/bjoc)

Table of contents:

General information……………………………………………………………………………………….S2
Experimental procedures…………………………………………………………………………………..S3
NMR spectra…………………………………………………………………………….…………………S8
Crystal structure determination……………………..…………………………………...……………………………….………S38

General information:

^{1}H, ^{13}C NMR, HMBC, HSQC, COSY 2D NMR spectra were acquired on a Bruker Avance-III HD 400 spectrometer in DMSO-d_6 or $CDCl_3$ using the solvent signal as an internal standard. IR spectra were recorded on a Perkin–Elmer Spectrum Two spectrometer from mulls in mineral oil. Melting points were measured on a Mettler Toledo MP90 apparatus. X-ray crystallography was performed on an Xcalibur Ruby diffractometer. Elemental analyses were carried out on a Vario MICRO Cube analyzer. The reaction conditions were optimized using HPLC [Hitachi Chromaster system; NUCLEODUR C18 Gravity column; grain size of 5 μm; acetonitrile–water as eluents; flow rate of 1.5 mL/min; Hitachi Chromaster 5430 diode array detector (wavelength range of 210–750 nm)] and UPLC [Waters ACQUITY UPLC I-Class system; Acquity UPLC BEH C18 column, grain size of 1.7 μm; acetonitrile–water as eluents; flow rate of 0.6 mL/min; ACQUITY UPLC PDA eλ Detector (wavelength range of 230–780 nm); Xevo TQD mass detector; electrospray ionization; positive ion detection; ion source temperature of 150 °C; capillary voltage of 3500–4000 V; cone voltage of 20–70 V; vaporizer temperature of 150–300 °C]. Thin-layer chromatography (TLC) was performed on Merck silica gel 60 F_{254} plates using EtOAc/toluene, 1:5 v/v as an eluent. Starting PBTs **2a**–**k** were obtained according to reported procedures[1] from oxalyl chloride (purchased from commercial vendors) and heterocyclic enamines (obtained according to reported procedures[1] from commercially available reagents). All solvents and reagents were purchased from commercial vendors and were used without additional purification.

References:

(1) (a) K. S. Bozdyreva, I. V. Smirnova, A. N. Maslivets, *Russ. J. Org. Chem.*, **2005**, *41*, 1081; (b) I. V. Mashevskaya, I. G. Mokrushin, K. S. Bozdyreva, A. N. Maslivets, *Russ. J. Org. Chem.*, **2011**, *47*, 253; (c) A. N. Maslivets, I. V. Mashevskaya, L. I. Smirnova, O. P. Krasnykh, S. N. Shurov, Yu. S. Andreichikov, *Zh. Org. Khim.*, **1992**, *28*, 2545; (d) E. E. Stepanova, A. V. Babenysheva, A. N. Maslivets, *Russ. J. Org. Chem.*, **2011**, *47*, 937.

Experimental procedures:

9-Aroyl-8-hydroxy-6-(2-hydroxyphenyl)-2-methylene-1-thia-3,6-diazaspiro[4.4]non-8-ene-4,7-diones 3a–k were not isolated. We only succeeded to isolate a relatively pure **3a** in order to determine if it existed in a form with an *exo*-methylene group.

9-Benzoyl-8-hydroxy-6-(2-hydroxyphenyl)-2-methylene-1-thia-3,6-diazaspiro[4.4]non-8-ene-4,7-dione (3a). A flask was charged with a solution of PBT **2a** (0.50 g, 1.6 mmol) and thioacetamide (0.12 g, 1.6 mmol) in 20 mL of acetone. When the violet color of the solution (characteristic of **2a**) disappeared, the solvent was evaporated. The residual solid was triturated with 20mL of dry toluene.

1*H*-Pyrrolo[3,2-*c*]pyridine-2,3-diones 4a–k; General procedure:
A flask was charged with a solution of PBT **2a**–**k** (1.00 g, 3.1 mmol) and thioacetamide (0.12 g, 1.6 mmol) in 20 mL of acetone, plugged with a piece of cotton and kept at room temperature in contact with the atmosphere air for 2–3 days. The orange precipitate formed was filtered off and washed with acetone to afford the desired 1*H*-pyrrolo[3,2-*c*]pyridine-2,3-dione **4a**–**k**.

1-(2-Hydroxyphenyl)-4-phenyl-6-sulfanylidene-5,6-dihydro-1*H*-pyrrolo[3,2-*c*]pyridine-2,3-dione (4a).
Orange solid; yield (0.29 g, 52%); mp 305–306 °C (decomp., from acetone).
^{1}H NMR (400 MHz, DMSO-d_6): δ = 13.39 (br s, 1 H), 9.90 (s, 1 H), 7.74 (m, 2 H), 7.65 (m, 1 H), 7.58 (m, 2 H), 7.40 (m, 1 H), 7.30 (m, 1 H), 7.09 (m, 1 H), 7.01 (m, 1 H), 6.20 (s, 1 H).
^{13}C NMR (100 MHz, DMSO-d_6): δ = 184.8, 175.9, 159.5, 153.1, 151.4, 150.5, 131.7, 131.0, 129.5 (2C), 128.8, 128.4, 128.0 (2C), 119.8, 118.7, 117.3, 108.8, 104.4.
IR (mineral oil, cm^{-1}): ṽ = 3440, 1733.
MS (ESI+): m/z calc. for $C_{19}H_{12}N_2O_3S+H^+$: 349.06; found: 349.10.
EA: Found: C, 65.6; H, 3.4; N, 8.0. Calc. for $C_{19}H_{12}N_2O_3S$: C, 65.5; H, 3.5; N, 8.0.

1-(5-Chloro-2-hydroxyphenyl)-4-phenyl-6-sulfanylidene-5,6-dihydro-1*H*-pyrrolo[3,2-*c*]pyridine-2,3-dione (4b).
Orange solid; yield (0.34 g, 55%); mp 333–335 °C (decomp., from acetone).
^{1}H NMR (400 MHz, DMSO-d_6): δ = 13.44 (br s, 1 H), 10.27 (s, 1 H), 7.73 (m, 2 H), 7.69 (m, 1 H), 7.58 (m, 2 H), 7.47–7.41 (m, 2 H), 7.11 (m, 1 H), 6.26 (s, 1 H).
^{13}C NMR (100 MHz, DMSO-d_6): δ = 184.7, 175.6, 159.4, 152.4, 150.9, 150.7, 131.8, 130.9, 129.5 (2C), 128.5, 128.2, 128.0 (2C), 122.6, 119.8, 118.9, 109.0, 104.3.
IR (mineral oil, cm^{-1}): ṽ = 3334, 1736.
MS (ESI+): m/z calc. for $C_{19}H_{11}ClN_2O_3S+H^+$: 383.03; found: 383.06.
EA: Found: C, 59.7; H, 2.9; N, 7.3. Calc. for $C_{19}H_{11}ClN_2O_3S$: C, 59.6; H, 2.9; N, 7.3.

4-(4-Ethoxyphenyl)-1-(2-hydroxyphenyl)-6-sulfanylidene-5,6-dihydro-1*H*-pyrrolo[3,2-*c*]pyridine-2,3-dione (4c).
Orange solid; yield (0.28 g, 45%); mp 308–310 °C (decomp., from acetone).
^{1}H NMR (400 MHz, DMSO-d_6): δ = 13.21 (s, 1 H), 9.90 (s, 1 H), 7.74 (m, 2 H), 7.40 (m, 1 H), 7.30 (m, 1 H), 7.10 (m, 3 H), 7.01 (m, 1 H), 6.16 (s, 1 H), 4.18 (q, *J* = 6.9 Hz, 2 H), 1.39 (t, *J* = 6.9 Hz, 3 H).
^{13}C NMR (100 MHz, DMSO-d_6): δ = 184.7, 175.9, 161.6, 159.6, 153.2, 151.6, 150.5, 131.7 (2C), 131.0, 128.9, 120.1, 119.8, 118.7, 117.3, 113.8 (2C), 108.4, 103.8, 63.5, 14.4.
IR (mineral oil, cm^{-1}): ṽ = 3370, 1733.
MS (ESI+): m/z calc. for $C_{21}H_{16}N_2O_4S+H^+$: 393.09; found: 393.10.
EA: Found: C, 64.4; H, 4.2; N, 7.1. Calc. for $C_{21}H_{16}N_2O_4S$: C, 64.3; H, 4.1; N, 7.1.

4-(4-Bromophenyl)-1-(2-hydroxyphenyl)-6-sulfanylidene-5,6-dihydro-1*H*-pyrrolo[3,2-*c*]pyridine-2,3-dione (4d).
Orange solid; yield (0.32 g, 47%); mp >350 °C (decomp., from acetone).
^{1}H NMR (400 MHz, DMSO-d_6): δ = 13.46 (br s, 1 H), 9.92 (s, 1 H), 7.79 (m, 2 H), 7.69 (m, 2 H), 7.40 (m, 1 H), 7.29 (m, 1 H), 7.09 (m, 1 H), 7.01 (m, 1 H), 6.21 (s, 1 H).
^{13}C NMR (100 MHz, DMSO-d_6): δ = 184.6, 176.0, 159.5, 153.1, 151.2, 149.2, 131.6 (2C), 131.1 (3C), 128.8, 127.4, 125.5, 119.8, 118.6, 117.3, 109.0, 104.6.
IR (mineral oil, cm^{-1}): ṽ = 3301, 1755, 1737.
MS (ESI+): m/z calc. for $C_{19}H_{11}BrN_2O_3S+H^+$: 426.98, 428.97; found: 427.02, 429.01.

EA: Found: C, 53.5; H, 2.7; N, 6.5. Calc. for $C_{19}H_{11}BrN_2O_3S$: C, 53.4; H, 2.6; N, 6.6.

1-(2-Hydroxyphenyl)-4-(4-methoxyphenyl)-6-sulfanylidene-5,6-dihydro-1*H*-pyrrolo[3,2-*c*]pyridine-2,3-dione (4e).
Orange solid; yield (0.28 g, 47%); mp 315–317 °C (decomp., from acetone).
^{1}H NMR (400 MHz, DMSO-d_6): δ = 13.22 (s, 1 H), 9.90 (s, 1 H), 7.75 (m, 2 H), 7.40 (m, 1 H), 7.30 (m, 1 H), 7.14–7.07 (m, 3 H), 7.01 (m, 1 H), 6.17 (s, 1 H), 3.89 (s, 3 H).
^{13}C NMR (100 MHz, DMSO-d_6): δ = 184.7, 175.9, 162.3, 159.6, 153.2, 151.6, 150.5, 131.6 (2C), 131.0, 128.9, 120.2, 119.8, 118.7, 117.3, 113.5 (2C), 108.4, 103.9, 55.5.
IR (mineral oil, cm^{-1}): ṽ = 3187, 1756, 1731.
MS (ESI+): m/z calc. for $C_{20}H_{14}N_2O_4S+H^+$: 379.08; found: 379.11.
EA: Found: C, 63.4; H, 3.75; N, 7.4. Calc. for $C_{20}H_{14}N_2O_4S$: C, 63.5; H, 3.7; N, 7.4.

4-(4-Chlorophenyl)-1-(2-hydroxyphenyl)-6-sulfanylidene-5,6-dihydro-1*H*-pyrrolo[3,2-*c*]pyridine-2,3-dione (4f).
Orange solid; yield (0.29 g, 48%); mp 324–326 °C (decomp., from acetone).
^{1}H NMR (400 MHz, DMSO-d_6): δ = 13.47 (br s, 1 H), 9.92 (s, 1 H), 7.77 (m, 2 H), 7.66 (m, 2 H), 7.41 (m, 1 H), 7.29 (m, 1 H), 7.09 (m, 1 H), 7.01 (m, 1 H), 6.22 (s, 1 H).
^{13}C NMR (100 MHz, DMSO-d_6): δ = 184.7, 176.0, 159.5, 153.1, 151.3, 149.1, 136.6, 131.5 (2C), 131.1, 128.8, 128.1 (2C), 127.0, 119.8, 118.7, 117.3, 109.0, 104.6.
IR (mineral oil, cm^{-1}): ṽ = 3304, 1756, 1737.
MS (ESI+): m/z calc. for $C_{19}H_{11}ClN_2O_3S+H^+$: 383.03; found: 383.03.
EA: Found: C, 59.6; H, 2.95; N, 7.2. Calc. for $C_{19}H_{11}ClN_2O_3S$: C, 59.6; H, 2.9; N, 7.3.

1-(2-Hydroxyphenyl)-4-(4-methylphenyl)-6-sulfanylidene-5,6-dihydro-1*H*-pyrrolo[3,2-*c*]pyridine-2,3-dione (4g).
Orange solid; yield (0.30 g, 51%); mp 325–327 °C (decomp., from acetone).
^{1}H NMR (400 MHz, DMSO-d_6): δ = 13.31 (s, 1 H), 9.90 (s, 1 H), 7.65 (m, 2 H), 7.43–7.38 (m, 3 H), 7.30 (m, 1 H), 7.09 (m, 1 H), 7.01 (m, 1 H), 6.19 (s, 1 H), 2.44 (s, 3 H).
^{13}C NMR (100 MHz, DMSO-d_6): δ = 184.7, 175.9, 159.6, 153.2, 151.5, 150.6, 142.1, 131.0, 129.5 (2C), 128.9, 128.6 (2C), 125.4, 119.8, 118.7, 117.3, 108.7, 104.2, 21.1.

IR (mineral oil, cm^{-1}): ṽ = 3187, 1756, 1733.
MS (ESI+): m/z calc. for $C_{20}H_{14}N_2O_3S+H^+$: 363.08; found: 363.11.
EA: Found: C, 66.2; H, 3.95; N, 7.7. Calc. for $C_{20}H_{14}N_2O_3S$: C, 66.3; H, 3.9; N, 7.7.

1-(5-Bromo-2-hydroxyphenyl)-4-phenyl-6-sulfanylidene-5,6-dihydro-1*H*-pyrrolo[3,2-*c*]pyridine-2,3-dione (4h).
Orange solid; yield (0.29 g, 42%); mp >350 °C (decomp., from acetone).
^{1}H NMR (400 MHz, DMSO-d_6): δ = 13.44 (s, 1 H), 10.30 (s, 1 H), 7.73 (m, 2 H), 7.65 (m, 1 H), 7.61–7.53 (m, 4 H), 7.07 (m, 1 H), 6.26 (s, 1 H).
^{13}C NMR (100 MHz, DMSO-d_6): δ = 184.7, 175.6, 159.4, 152.9, 150.9, 150.7, 133.8, 131.8, 131.3, 129.5 (2C), 128.2, 128.0 (2C), 120.2, 119.4, 109.7, 109.0, 104.3.
IR (mineral oil, cm^{-1}): ṽ = 3314, 1733.
MS (ESI+): m/z calc. for $C_{19}H_{11}BrN_2O_3S+H^+$: 426.98, 428.97; found: 427.00, 428.99.
EA: Found: C, 53.4; H, 2.5; N, 6.6. Calc. for $C_{19}H_{11}BrN_2O_3S$: C, 53.4; H, 2.6; N, 6.6.

1-(2-Hydroxy-5-methylphenyl)-4-phenyl-6-sulfanylidene-5,6-dihydro-1*H*-pyrrolo[3,2-*c*]pyridine-2,3-dione (4i).
Orange solid; yield (0.26 g, 44%); mp >350 °C (decomp., from acetone).
^{1}H NMR (400 MHz, DMSO-d_6): δ = 13.38 (br s, 1 H), 9.64 (s, 1 H), 7.73 (m, 2 H), 7.66 (m, 1 H), 7.59 (m, 2 H), 7.21 (m, 1 H), 7.10 (m, 1 H), 6.68 (m, 1 H), 6.21 (s, 1 H), 2.28 (s, 3 H).
^{13}C NMR (100 MHz, DMSO-d_6): δ = 184.6, 176.0, 159.5, 151.4, 150.7, 150.4, 131.7, 131.6, 129.5 (2C), 128.8, 128.7, 128.3, 128.0 (2C), 118.3, 117.2, 108.9, 104.4, 19.8.
IR (mineral oil, cm^{-1}): ṽ = 3181, 1754, 1738.
MS (ESI+): m/z calc. for $C_{20}H_{14}N_2O_3S+H^+$: 363.08; found: 363.10.
EA: Found: C, 66.2; H, 3.9; N, 7.6. Calc. for $C_{20}H_{14}N_2O_3S$: C, 66.3; H, 3.9; N, 7.7.

1-(2-Hydroxy-4-nitrophenyl)-4-phenyl-6-sulfanylidene-5,6-dihydro-1*H*-pyrrolo[3,2-*c*]pyridine-2,3-dione (4j).
Orange solid; yield (0.24 g, 38%); mp >350 °C (decomp., from acetone).
^{1}H NMR (400 MHz, DMSO-d_6): δ = 13.48 (br s, 1 H), 11.09 (s, 1 H), 7.88 (m, 2 H), 7.74 (m, 2 H), 7.68–7.56 (m, 4 H), 6.34 (s, 1 H).
^{13}C NMR (100 MHz, DMSO-d_6): δ = 184.8, 175.3, 159.1, 154.0, 150.8, 150.4, 148.8, 131.8, 130.3, 129.5 (2C), 128.2, 128.0 (2C), 124.5, 114.6, 112.0, 109.2, 104.4.

IR (mineral oil, cm^{-1}): ṽ = 3175, 1731.
MS (ESI+): m/z calc. for $C_{19}H_{11}N_3O_5S+H^+$: 394.05; found: 394.08.
EA: Found: C, 58.1; H, 2.9; N, 10.75. Calc. for $C_{19}H_{11}N_3O_5S$: C, 58.0; H, 2.8; N, 10.7.

4-(3-Bromophenyl)-1-(5-chloro-2-hydroxyphenyl)-6-sulfanylidene-5,6-dihydro-1*H*-pyrrolo[3,2-*c*]pyridine-2,3-dione (4k).
Orange solid; yield (0.31 g, 42%); mp 270 °C (decomp., from acetone).
^{1}H NMR (400 MHz, DMSO-d_6): δ = 13.55 (s, 1 H), 10.30 (s, 1 H), 7.94 (m, 1 H), 7.85 (m, 1 H), 7.73 (m, 1 H), 7.54 (m, 1 H), 7.46 (m, 1 H), 7.40 (m, 1 H), 7.11 (m, 1 H), 6.28 (s, 1 H).
^{13}C NMR (100 MHz, DMSO-d_6): δ = 184.7, 175.7, 159.3, 152.4, 150.6, 148.7, 134.4, 132.1, 130.9, 130.2, 130.1, 128.6, 128.4, 122.6, 121.0, 119.7, 118.9, 109.3, 104.6.
IR (mineral oil, cm^{-1}): ṽ = 3175, 1752, 1741, 1723.
MS (ESI+): m/z calc. for $C_{19}H_{10}BrClN_2O_3S+H^+$: 460.94, 462.93; found: 460.96, 462.94.
EA: Found: C, 49.5; H, 2.1; N, 6.15. Calc. for $C_{19}H_{10}BrClN_2O_3S$: C, 49.4; H, 2.2; N, 6.1.

3-Benzoyl-3a-((9-benzoyl-8-hydroxy-6-(2-hydroxyphenyl)-4,7-dioxo-3-phenyl-1-thia-3,6-diazaspiro[4.4]non-8-en-2-ylidene)methyl)-2-hydroxy-1*H*-pyrrolo[2,1-*c*][1,4]benzoxazine-1,4(3a*H*)-dione (5).
A flask was charged with a solution of PBT **2a** (0.50 g, 1.6 mmol) and phenylthioacetamide (0.24 g, 1.6 mmol) in 20 mL of acetone, plugged with a piece of cotton and kept at room temperature for 12 h. The yellow precipitate formed was filtered off to afford the compound **5**.
Yellow solid; yield (0.50 g, 40%); mp 217–219 °C (decomp., from acetone).
^{1}H NMR (400 MHz, $CDCl_3$): δ = 4.48 (s, 1 H), 6.70–7.83 (m, 26 H).
^{13}C NMR was not acquired due to poor solubility of the compound **5** in $CDCl_3$ (our attempts to acquire ^{13}C NMR in polar solvents (DMSO-d_6, CD_3OD, CD_3CN) were not successful because the compound **5** decomposed during acquisition of the spectra).
IR (mineral oil, cm^{-1}): ṽ = 3325, 1788, 1734, 1717, 1696, 1665.
MS (ESI+): m/z calc. for $C_{44}H_{27}N_3O_{10}S–CO+H^+$: 762.15; found: 762.10.
EA: Found: C, 67.0; H, 3.5; N, 5.2. Calc. for $C_{44}H_{27}N_3O_{10}S$: C, 66.9; H, 3.5; N, 5.3.

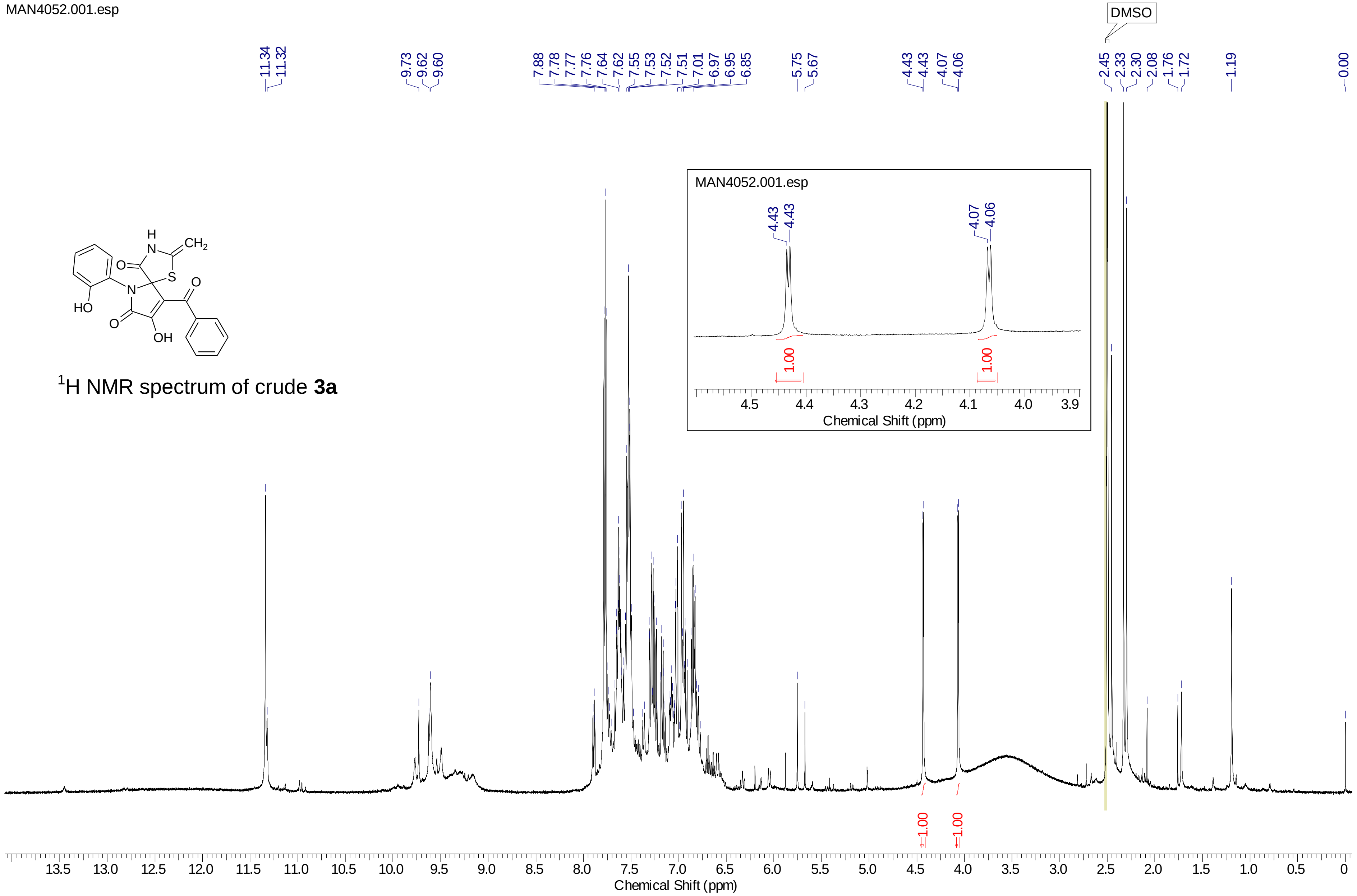

^{1}H NMR spectrum of crude **3a**

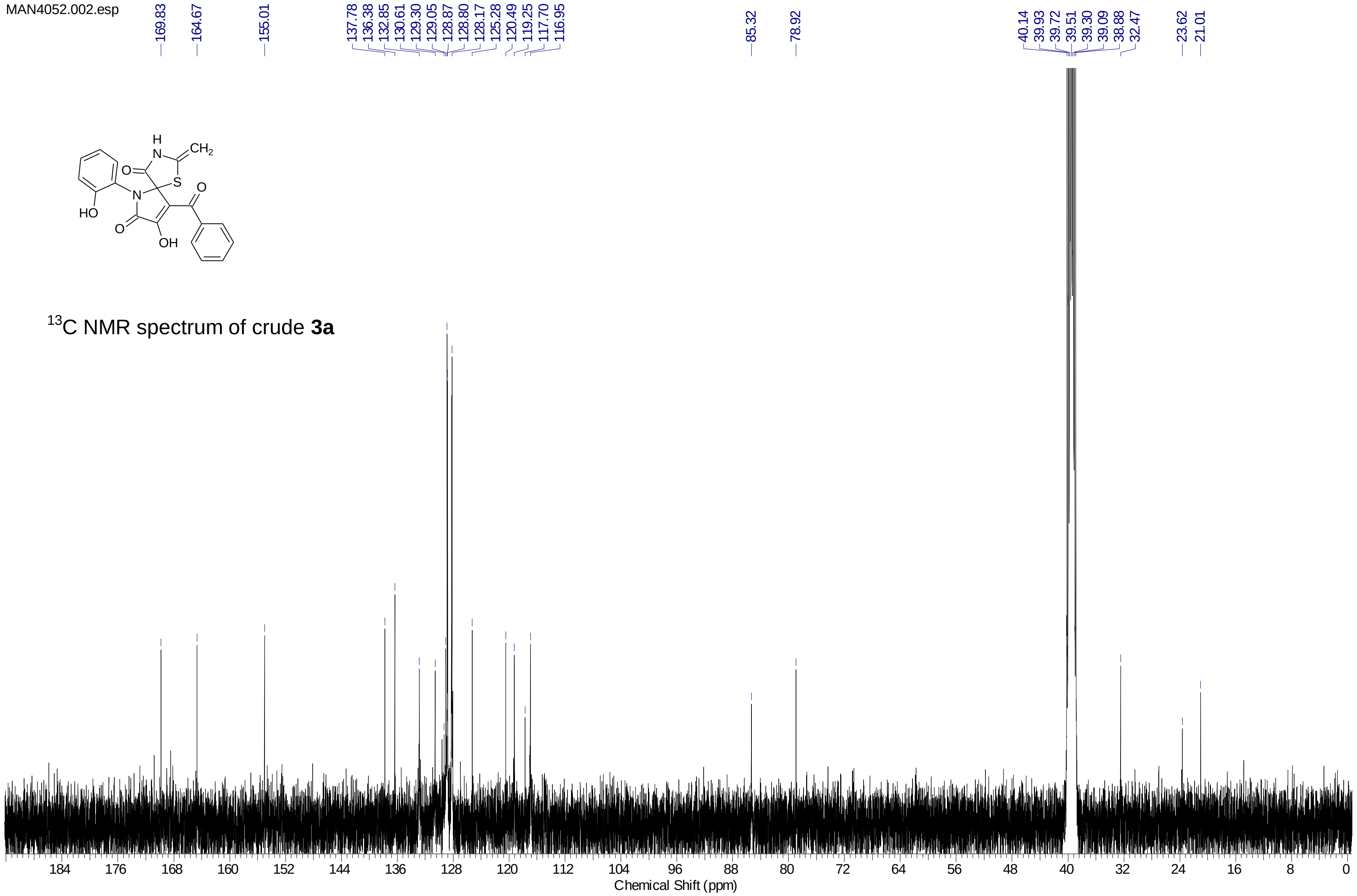

^{13}C NMR spectrum of crude **3a**

MAN4052.003.esp

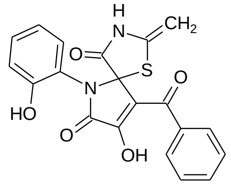

DEPT135 NMR spectrum of crude **3a**

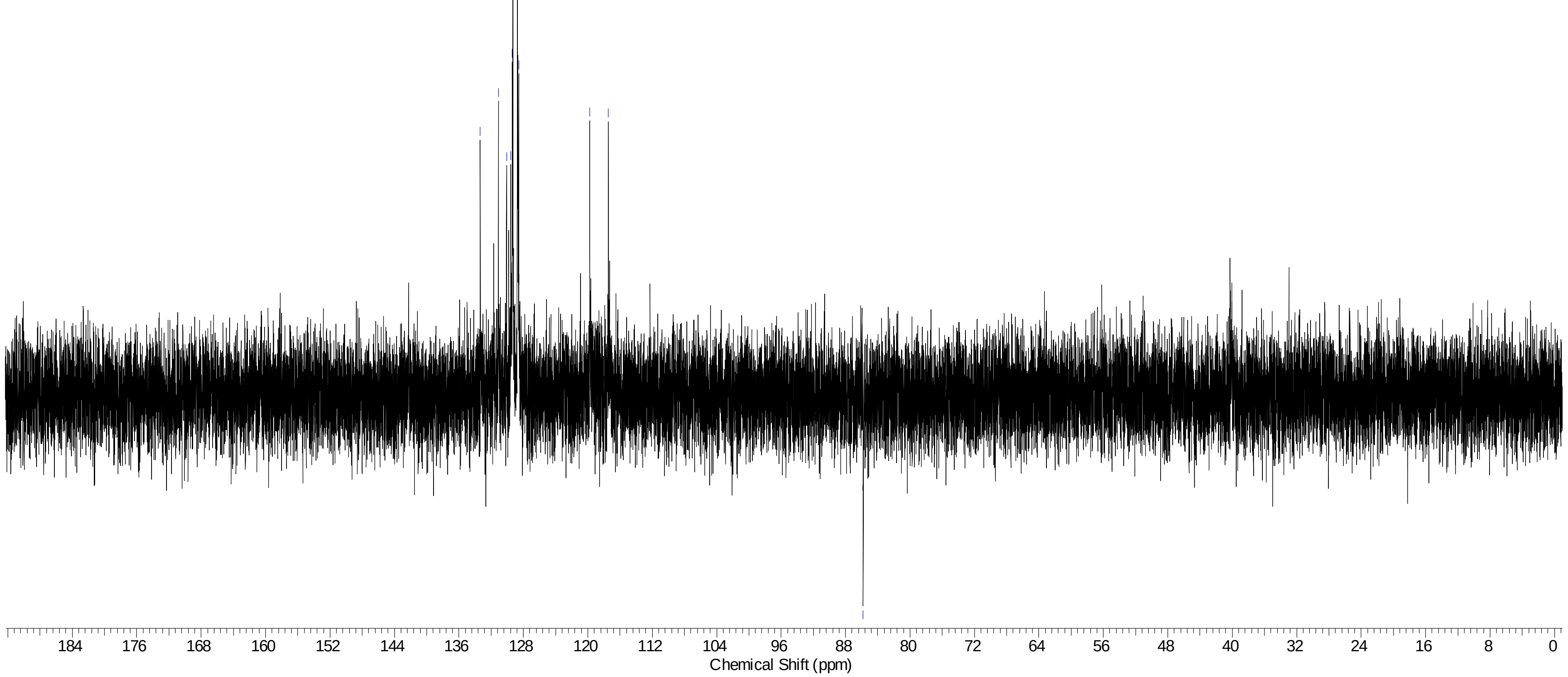

^{1}H NMR of **4a**

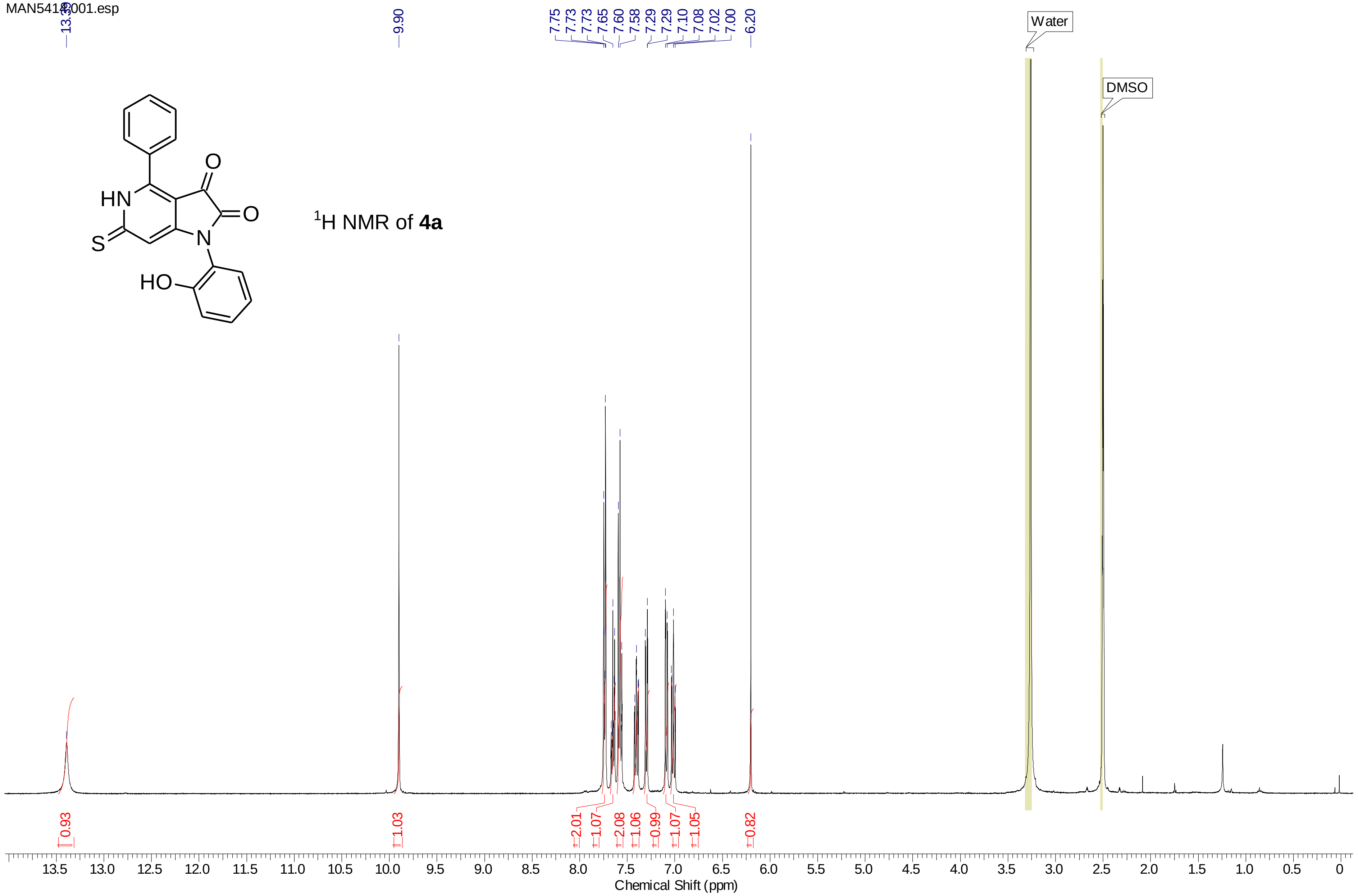
13.39
9.90
7.75
7.73
7.73
7.65
7.60
7.58
7.29
7.29
7.10
7.08
7.02
7.00
6.20
Water
DMSO
0.93
1.03
2.01
1.07
2.08
1.06
0.99
1.07
1.05
0.82
13.5 13.0 12.5 12.0 11.5 11.0 10.5 10.0 9.5 9.0 8.5 8.0 7.5 7.0 6.5 6.0 5.5 5.0 4.5 4.0 3.5 3.0 2.5 2.0 1.5 1.0 0.5 0
Chemical Shift (ppm)

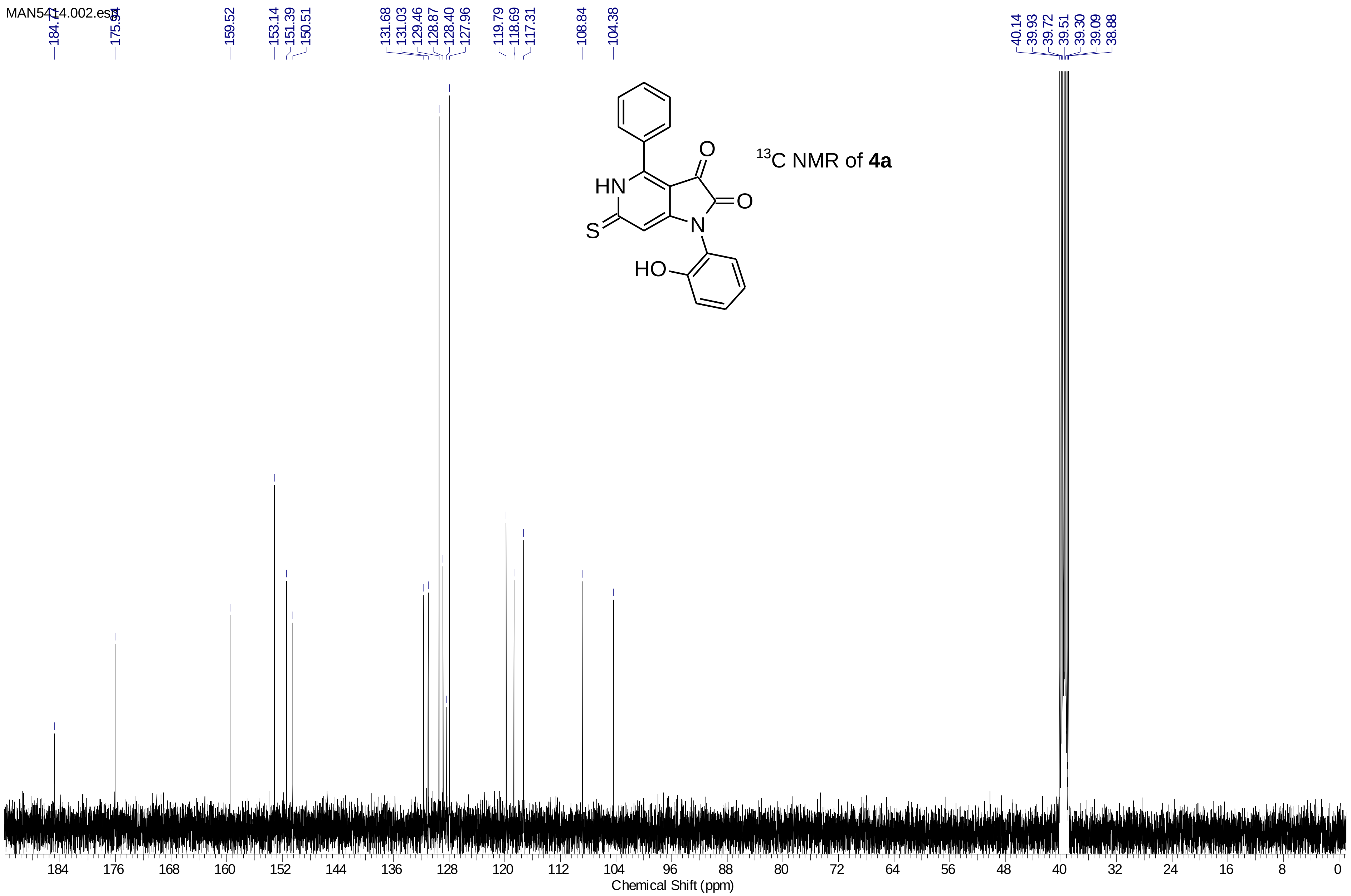

MAN5414.002.esp
184.71
175.94
159.52
153.14
151.39
150.51
131.68
131.03
129.46
128.87
128.40
127.96
119.79
118.69
117.31
108.84
104.38
40.14
39.93
39.72
39.51
39.30
39.09
38.88
HN
S
O
O
N
HO
184 176 168 160 152 144 136 128 120 112 104 96 88 80 72 64 56 48 40 32 24 16 8 0
Chemical Shift (ppm)

^{13}C NMR of **4a**

MAN4550.001.esp

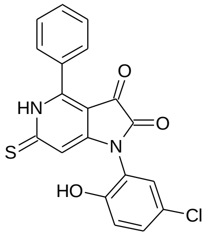

^{1}H NMR of **4b**

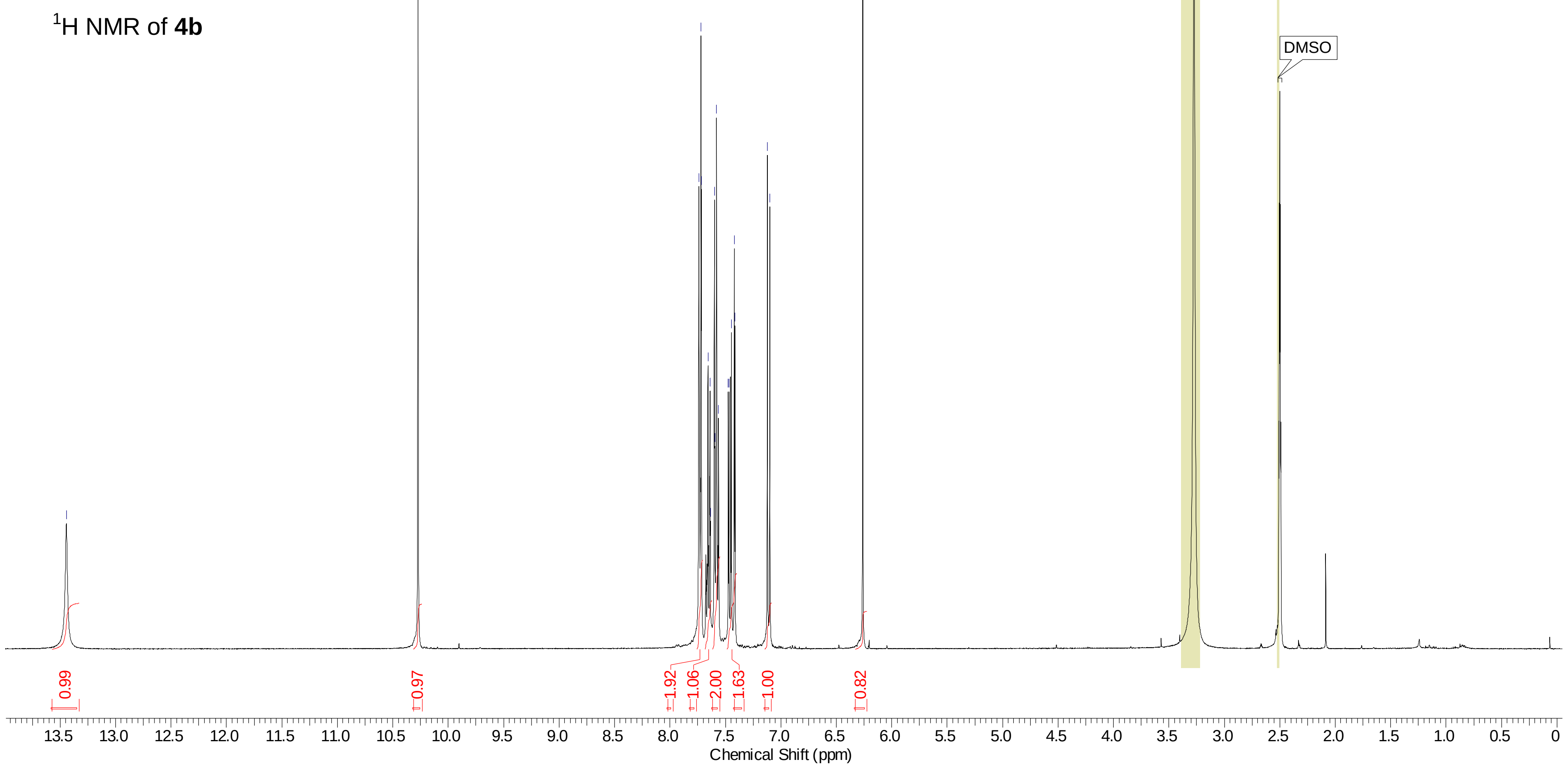

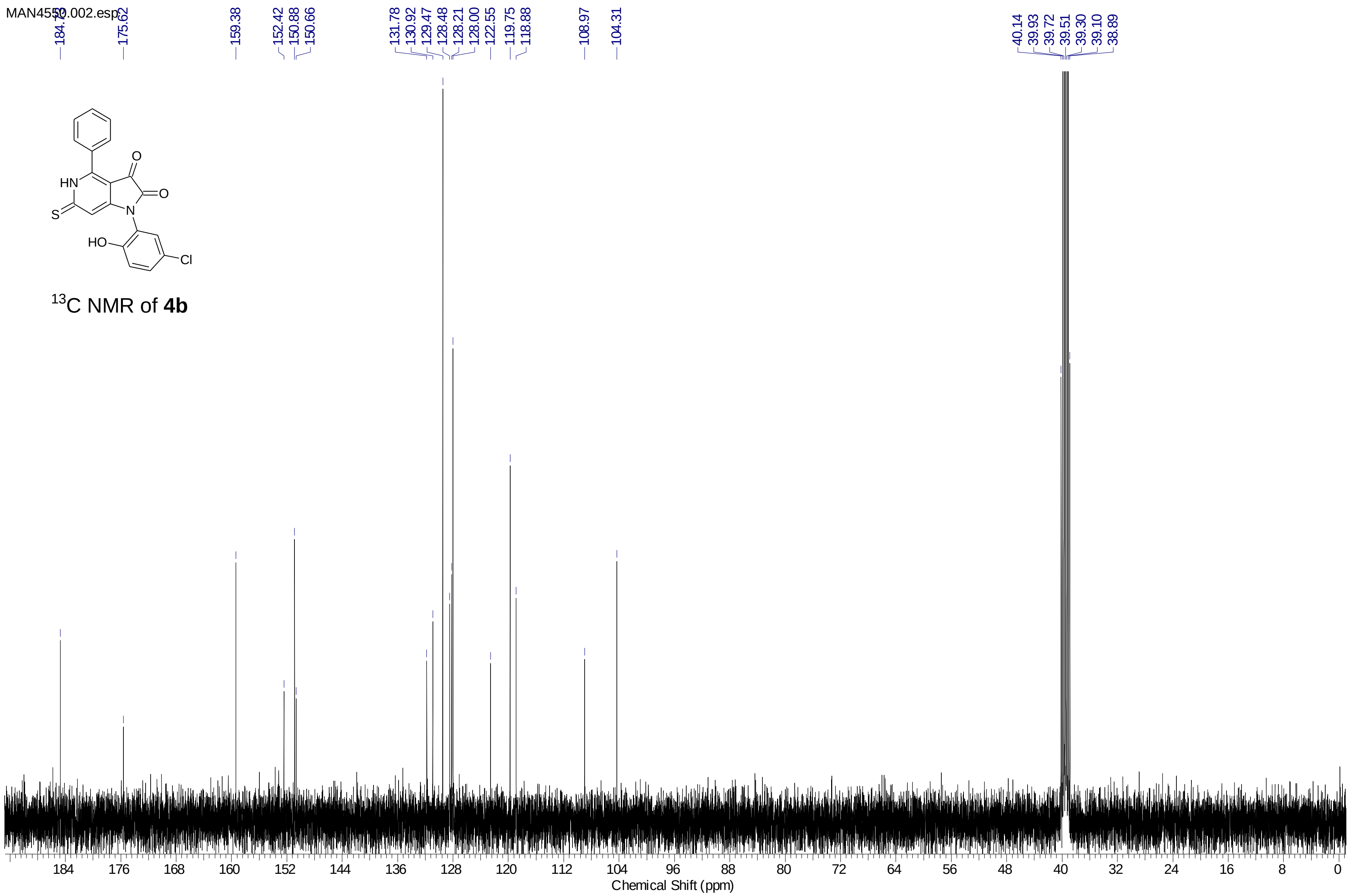
MAN4550.002.esp
184.70
175.62
159.38
152.42
150.88
150.66
131.78
130.92
129.47
128.48
128.21
128.00
122.55
119.75
118.88
108.97
104.31
40.14
39.93
39.72
39.51
39.30
39.10
38.89
O
HN
O
N
S
HO
Cl
^{13}C NMR of **4b**
184 176 168 160 152 144 136 128 120 112 104 96 88 80 72 64 56 48 40 32 24 16 8 0
Chemical Shift (ppm)

MAN5394.001.esp

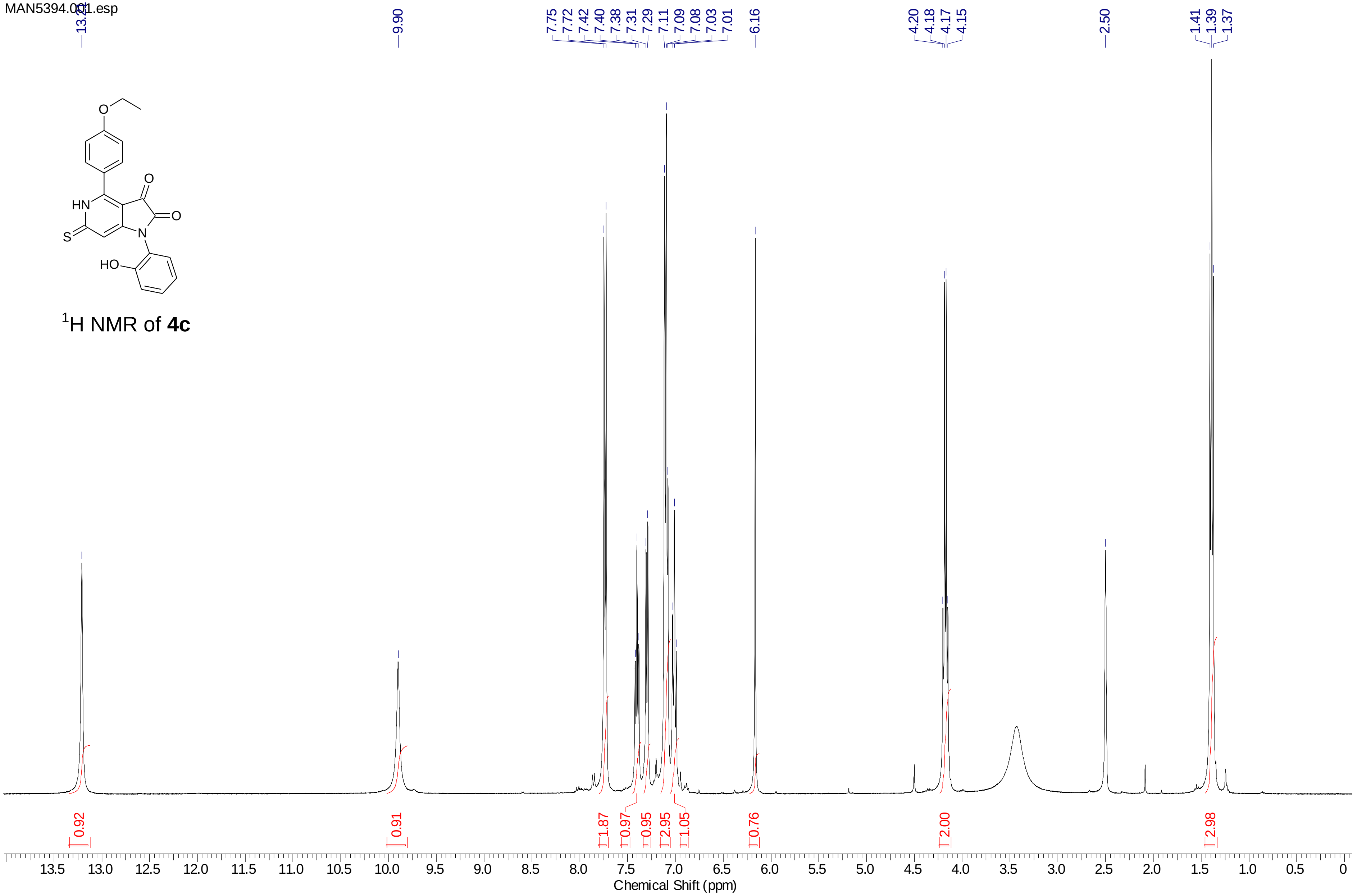

^{1}H NMR of **4c**

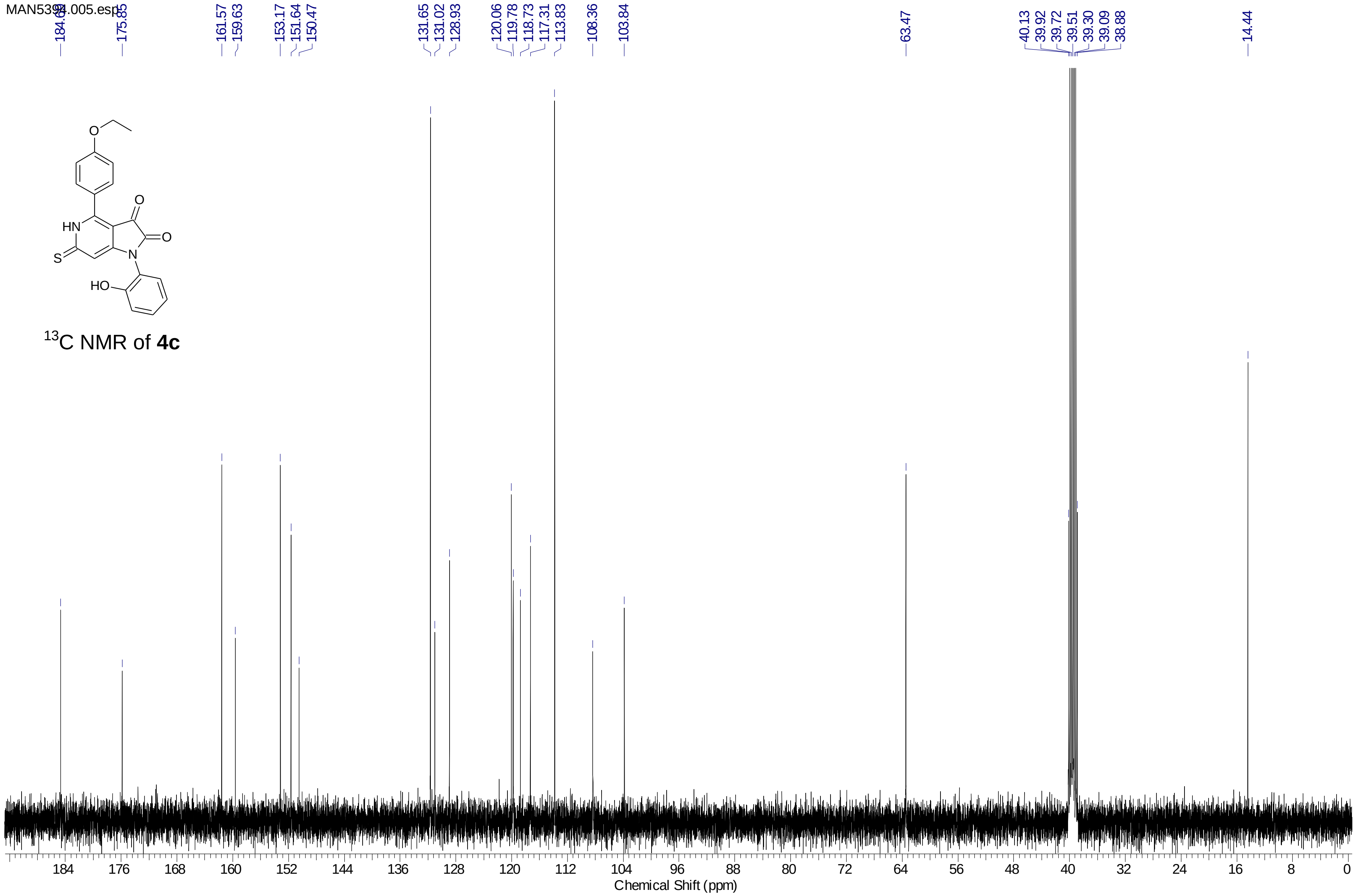

161.57
159.63
153.17
151.64
150.47
131.65
131.02
128.93
120.06
119.78
118.73
117.31
113.83
108.36
103.84
63.47
40.13
39.92
39.72
39.51
39.30
39.09
38.88
14.44
O
HN
N
S
HO
^{13}C NMR of **4c**
184 176 168 160 152 144 136 128 120 112 104 96 88 80 72 64 56 48 40 32 24 16 8 0
Chemical Shift (ppm)

MAN4949.003.esp

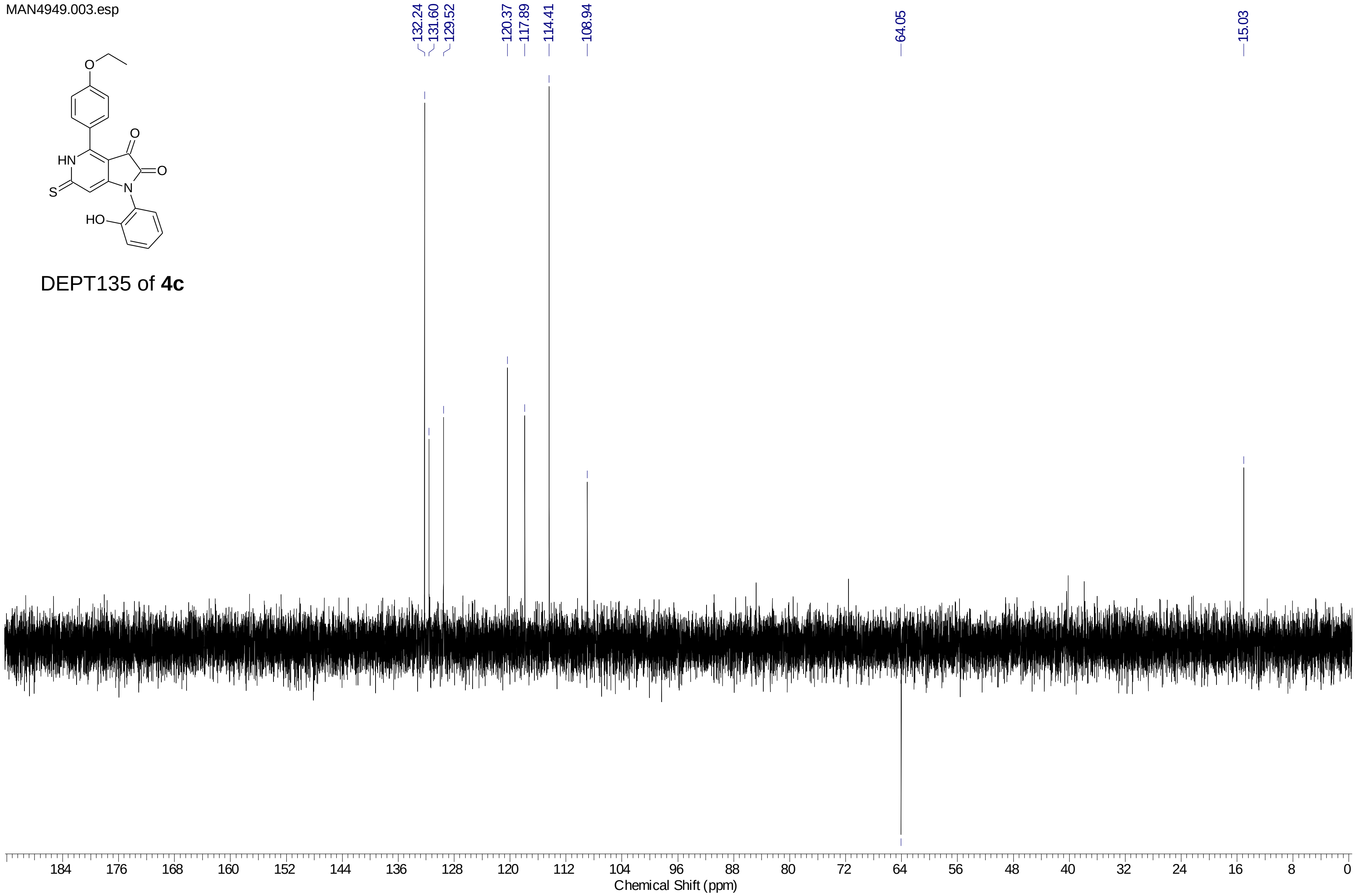

DEPT135 of **4c**

MAN4949.004.001.2rr.esp

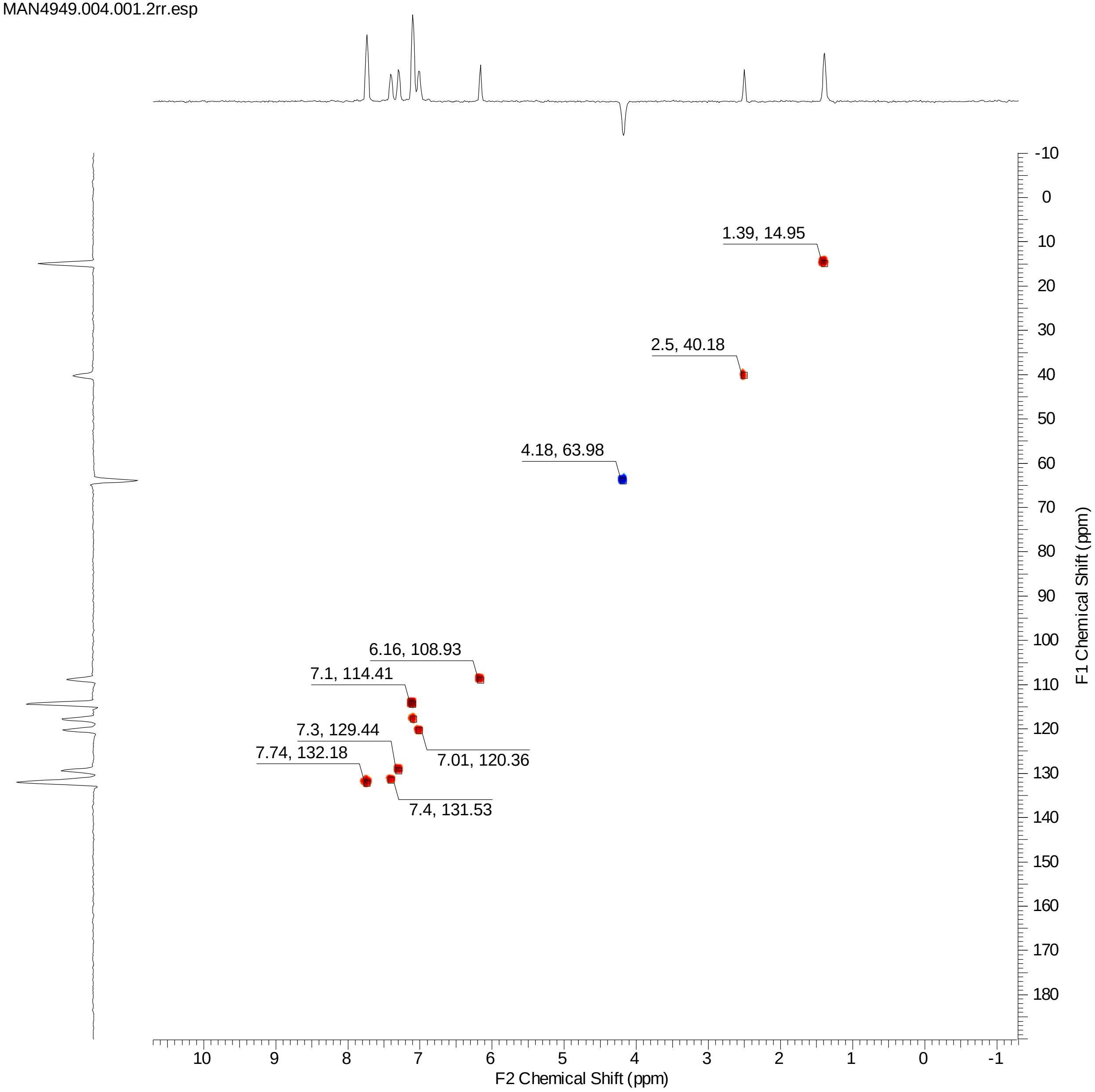

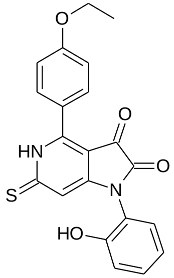

HMBC of **4c**

MAN4949.005.001.2rr.esp

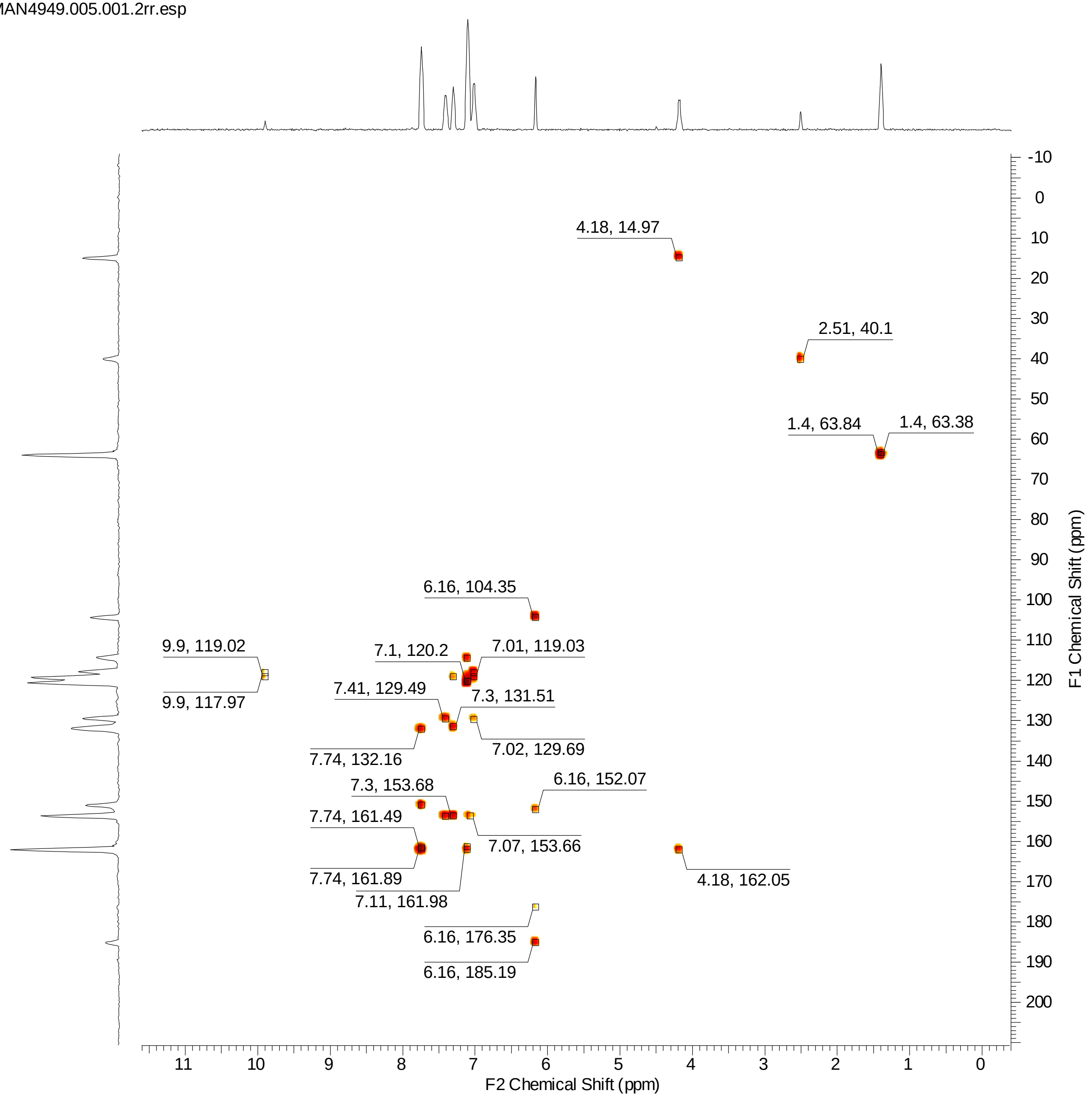

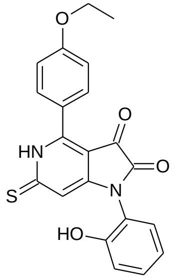

HSQC of **4c**

MAN4949.007.001.2rr.esp

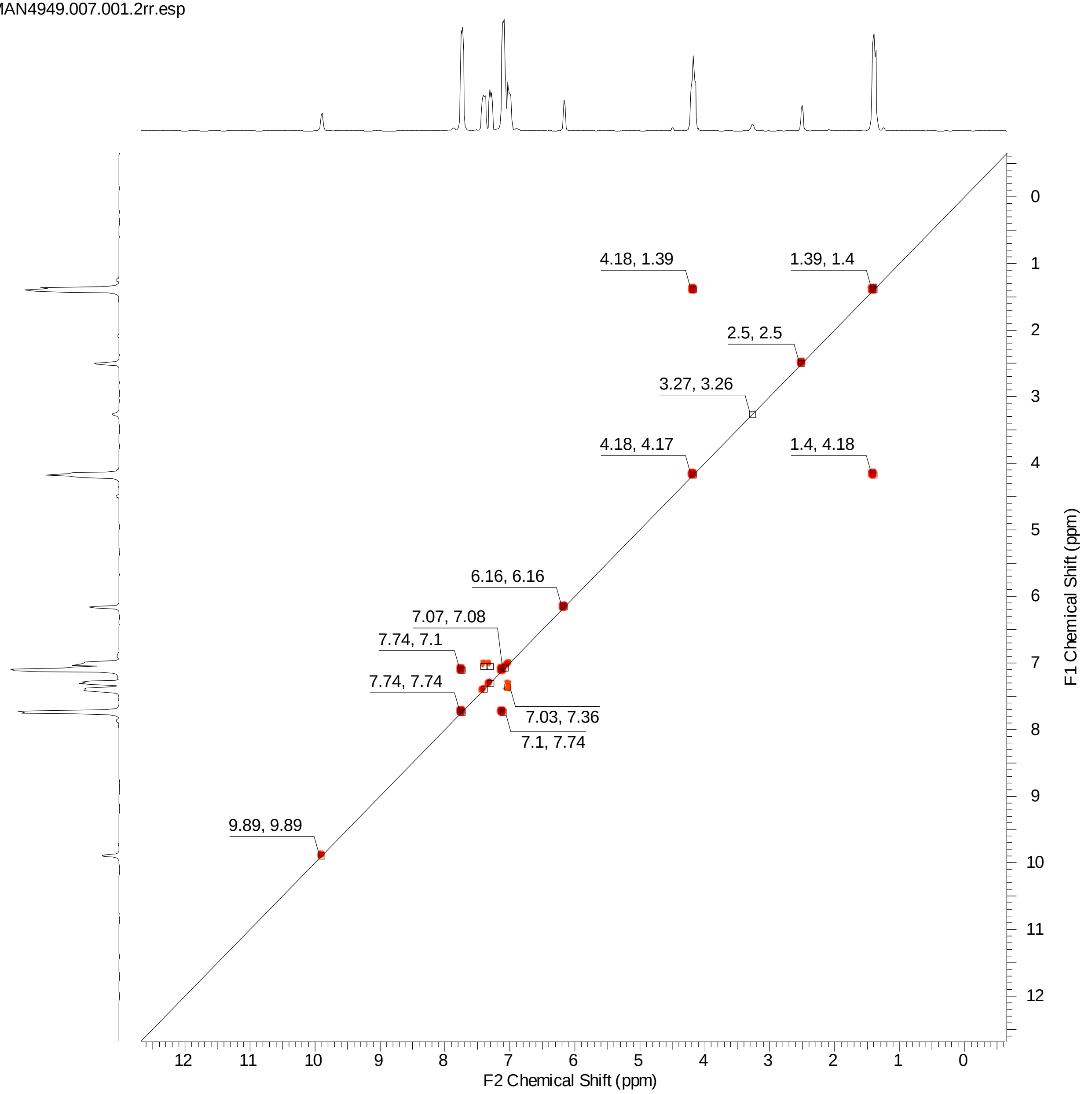

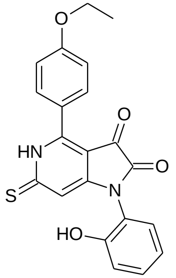

COSY of **4c**

MAN5399.001.esp

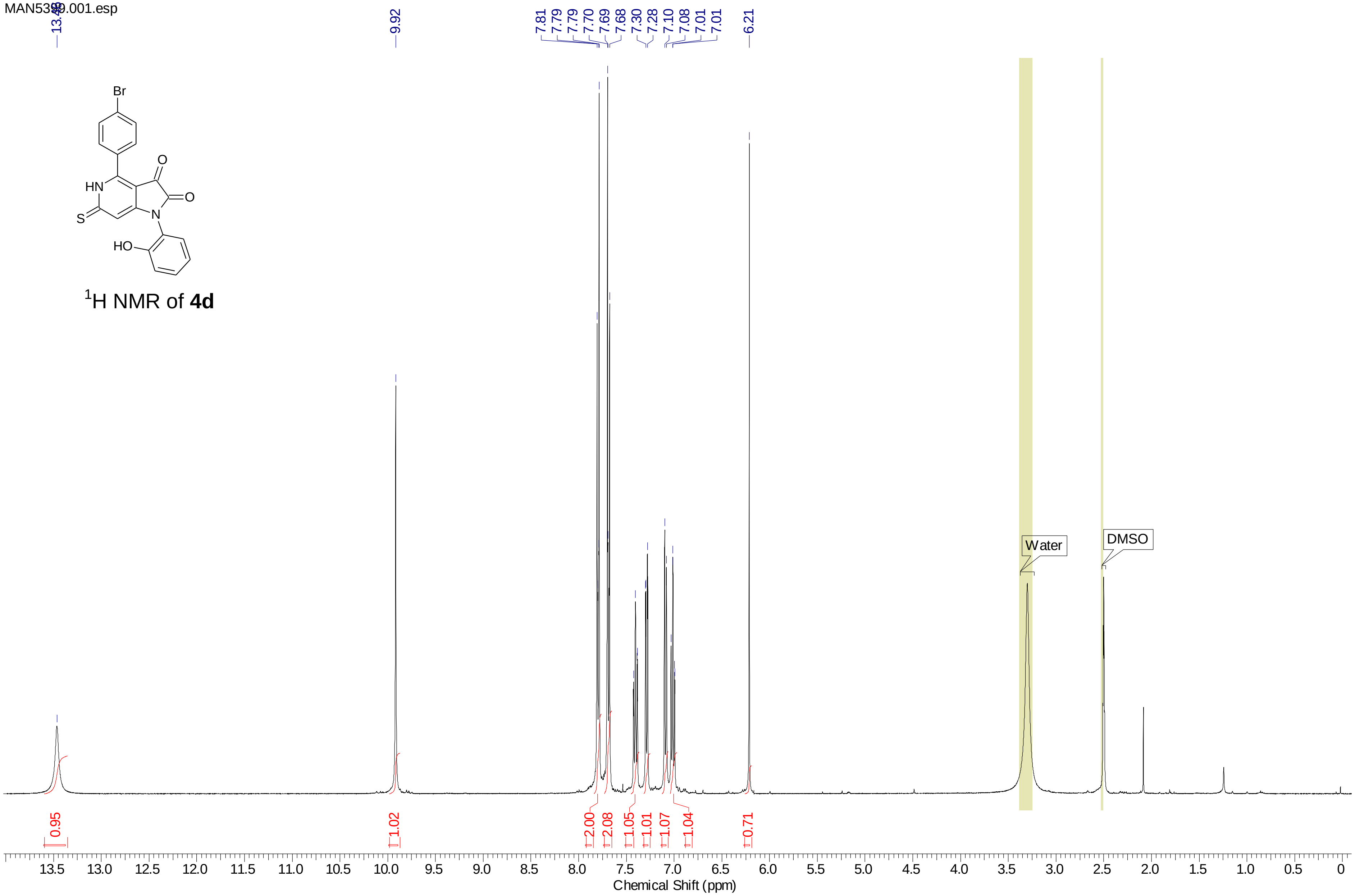

^{1}H NMR of **4d**

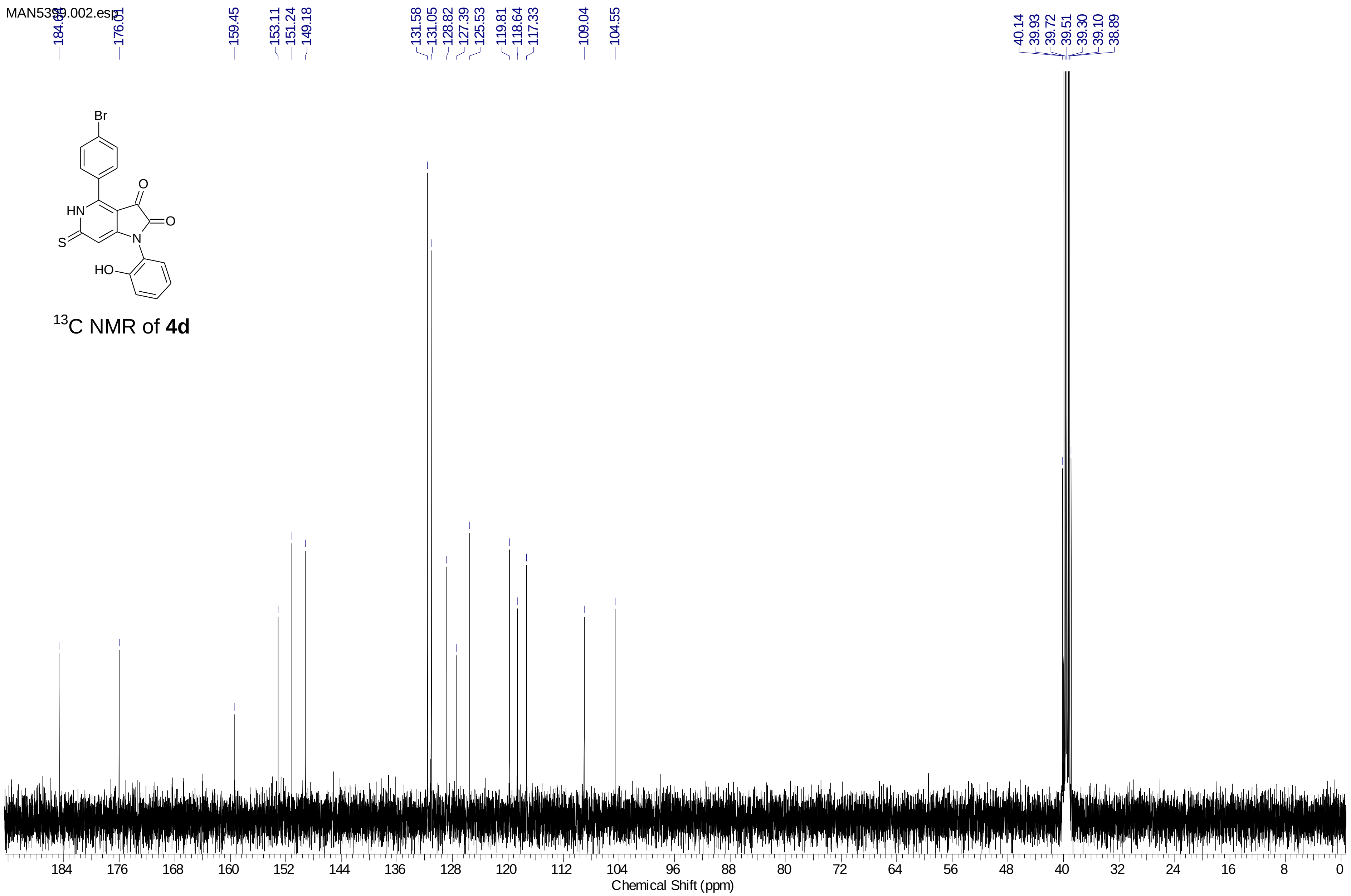
MAN5399.002.esp
184.69
176.01
159.45
153.11
151.24
149.18
131.58
131.05
128.82
127.39
125.53
119.81
118.64
117.33
109.04
104.55
40.14
39.93
39.72
39.51
39.30
39.10
38.89
Br
O
HN
O
S
N
HO
^{13}C NMR of **4d**
Chemical Shift (ppm)

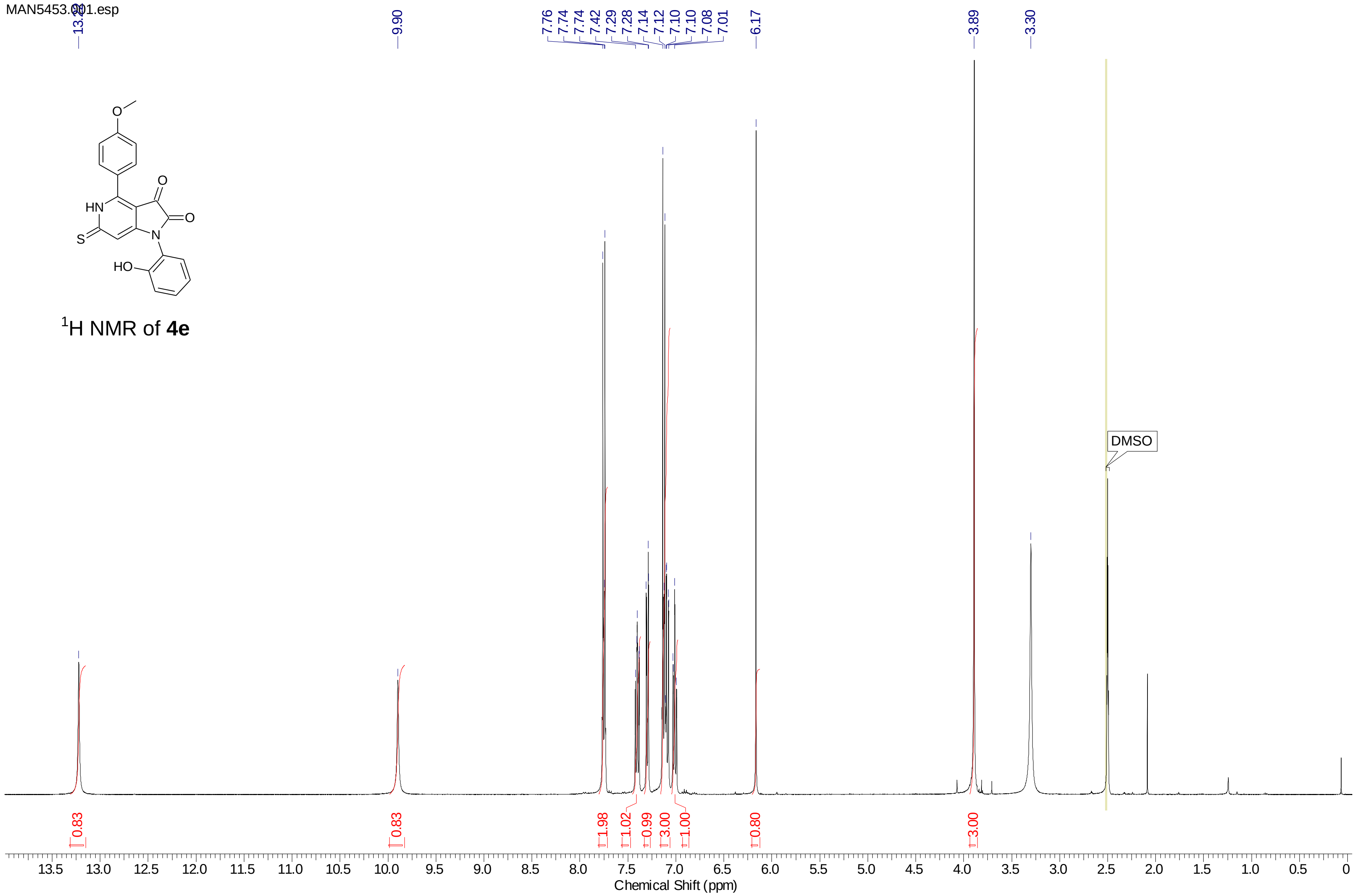
MAN5453.001.esp
13.20
9.90
7.76
7.74
7.74
7.42
7.29
7.28
7.14
7.12
7.10
7.10
7.08
7.01
6.17
3.89
3.30
^{1}H NMR of **4e**
DMSO
0.83
0.83
1.98
1.02
0.99
3.00
1.00
0.80
3.00
Chemical Shift (ppm)

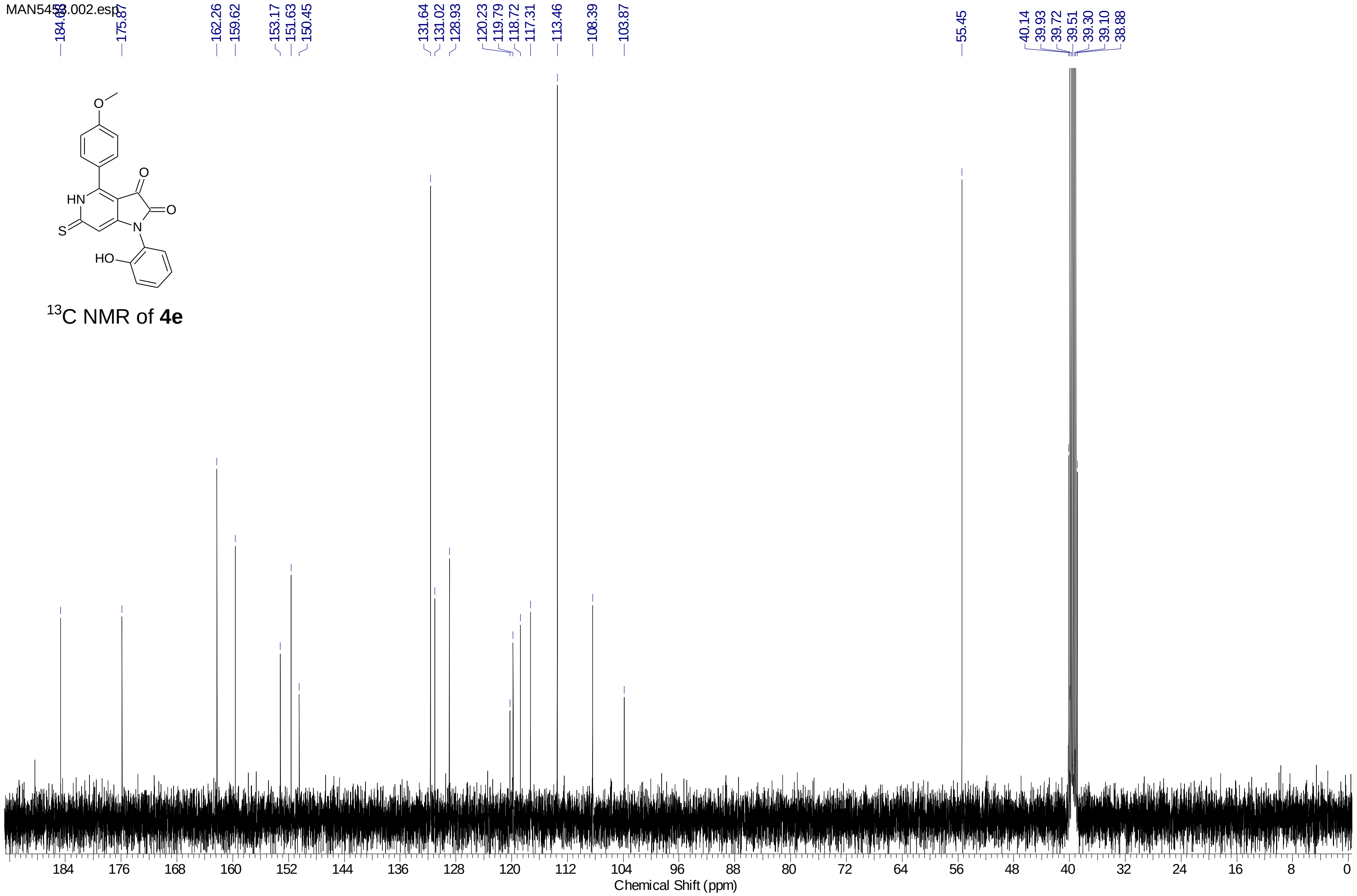
MAN5453.002.esp
184.63
175.87
162.26
159.62
153.17
151.63
150.45
131.64
131.02
128.93
120.23
119.79
118.72
117.31
113.46
108.39
103.87
55.45
40.14
39.93
39.72
39.51
39.30
39.10
38.88
O
HN
O
O
S
N
HO
13C NMR of 4e
184 176 168 160 152 144 136 128 120 112 104 96 88 80 72 64 56 48 40 32 24 16 8 0
Chemical Shift (ppm)

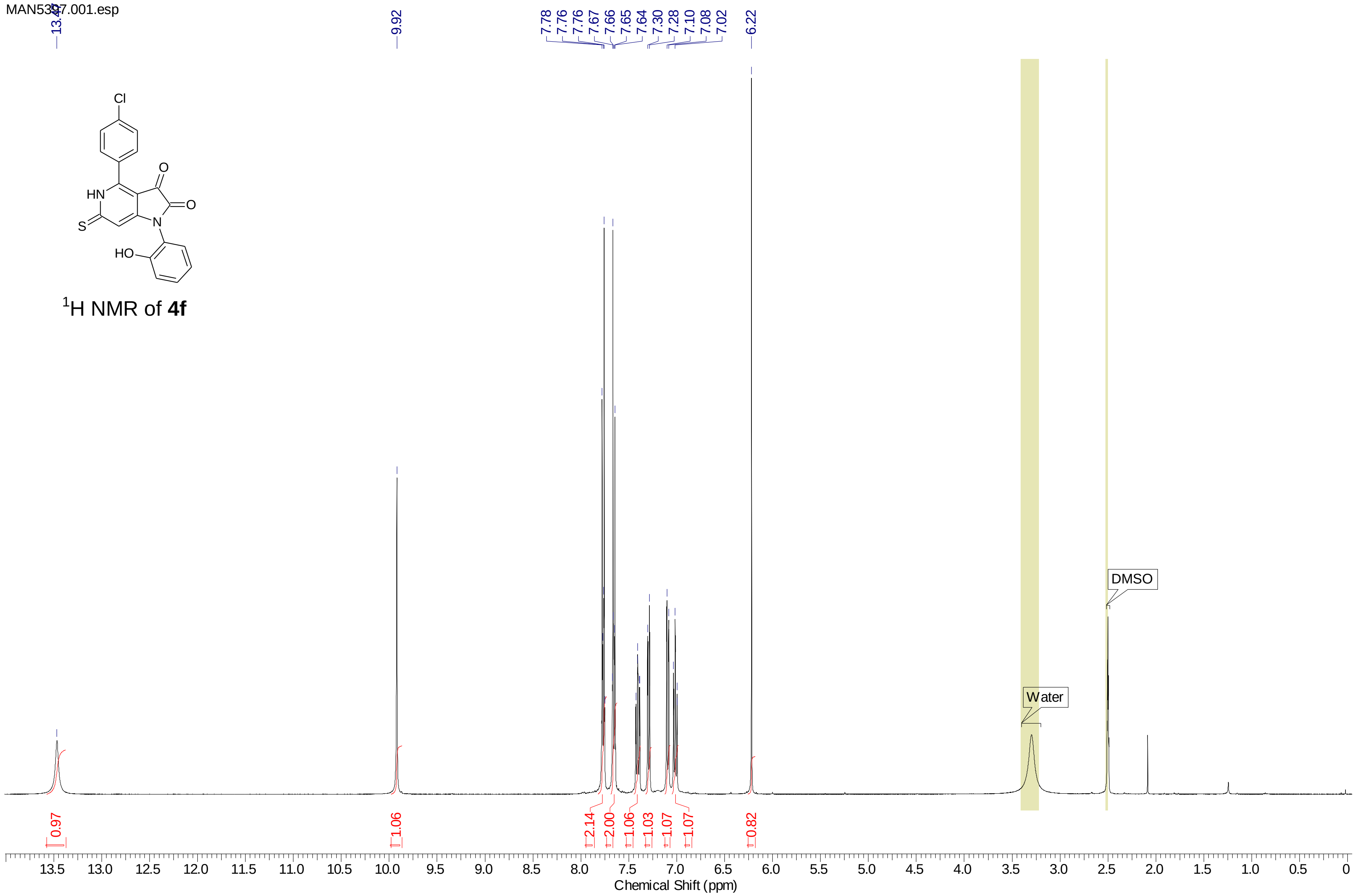

13.47
9.92
7.78
7.76
7.76
7.67
7.66
7.65
7.64
7.30
7.28
7.10
7.08
7.02
6.22
Cl
O
HN
O
N
S
HO
DMSO
Water
0.97
1.06
2.14
2.00
1.06
1.03
1.07
1.07
0.82
13.5 13.0 12.5 12.0 11.5 11.0 10.5 10.0 9.5 9.0 8.5 8.0 7.5 7.0 6.5 6.0 5.5 5.0 4.5 4.0 3.5 3.0 2.5 2.0 1.5 1.0 0.5 0
Chemical Shift (ppm)

^{1}H NMR of **4f**

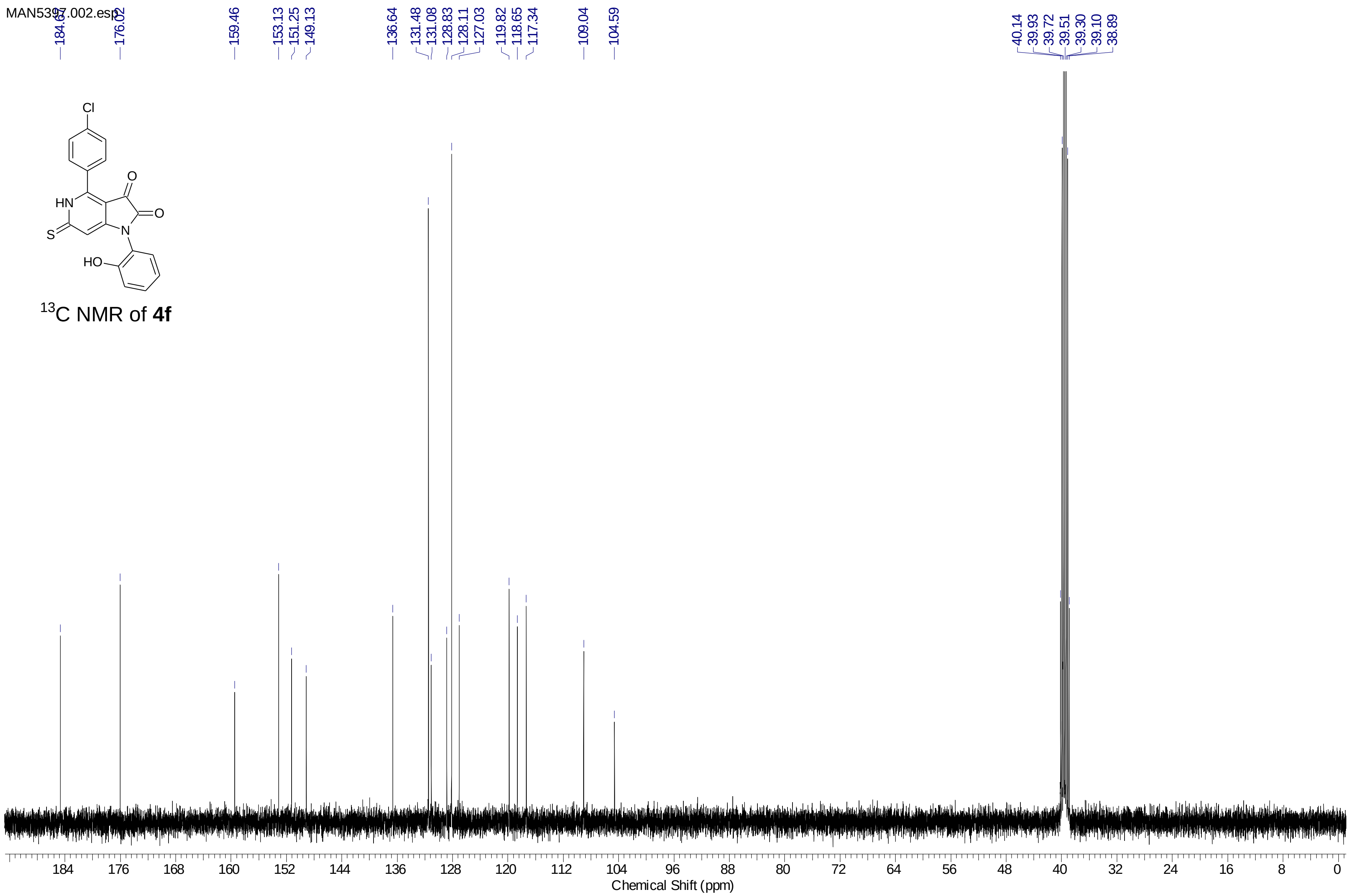
MAN5397.002.esp
184.63
176.02
159.46
153.13
151.25
149.13
136.64
131.48
131.08
128.83
128.11
127.03
119.82
118.65
117.34
109.04
104.59
40.14
39.93
39.72
39.51
39.30
39.10
38.89
Cl
O
HN
O
S
N
HO
184
176
168
160
152
144
136
128
120
112
104
96
88
80
72
64
56
48
40
32
24
16
8
0
Chemical Shift (ppm)

^{13}C NMR of **4f**

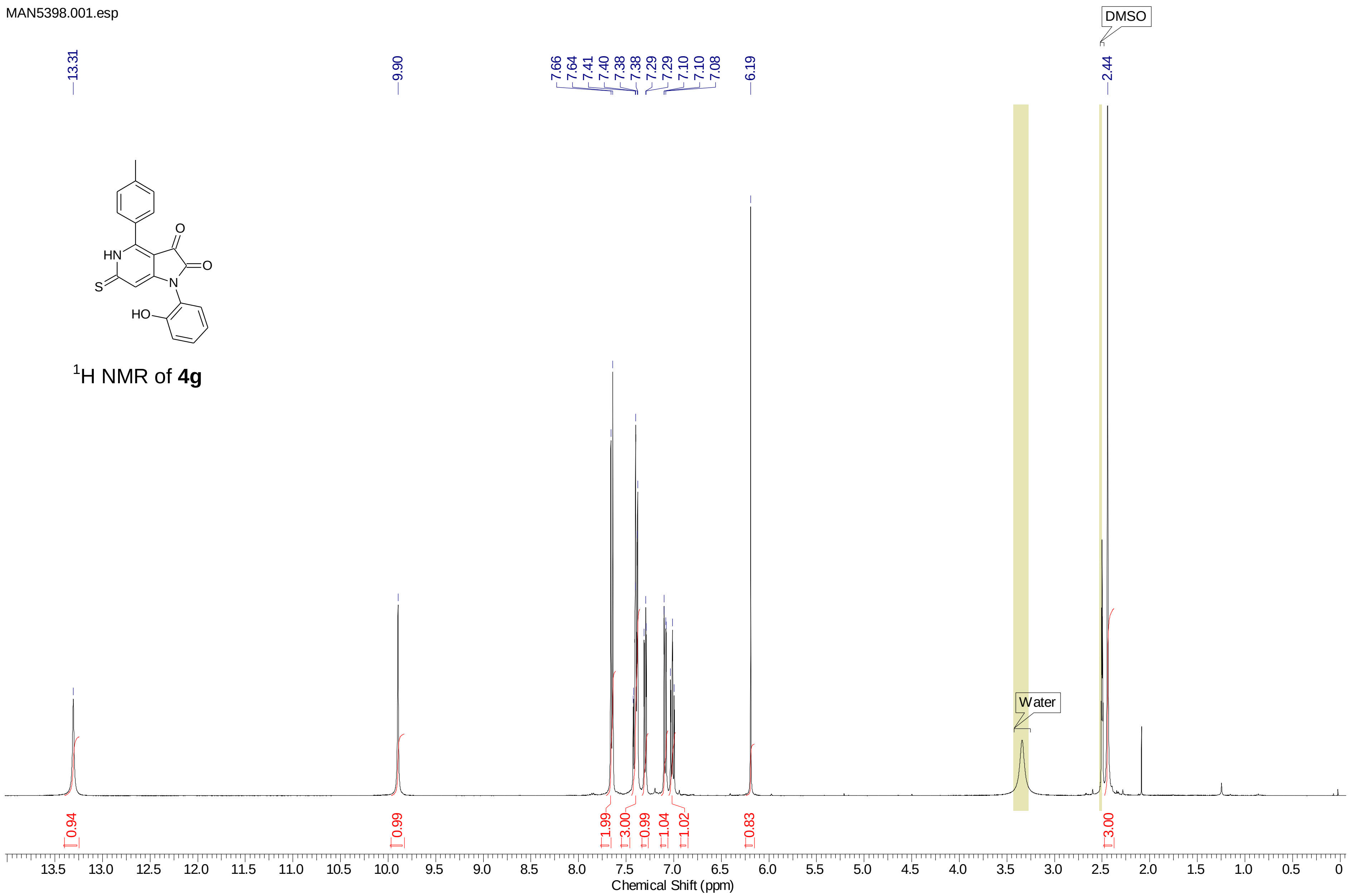

MAN5398.001.esp
DMSO
13.31
9.90
7.66 7.64 7.41 7.40 7.38 7.38 7.29 7.29 7.10 7.10 7.08
6.19
2.44
HN
S
O
O
N
HO
Water
0.94
0.99
1.99 3.00 0.99 1.04 1.02
0.83
3.00
Chemical Shift (ppm)

^{1}H NMR of **4g**

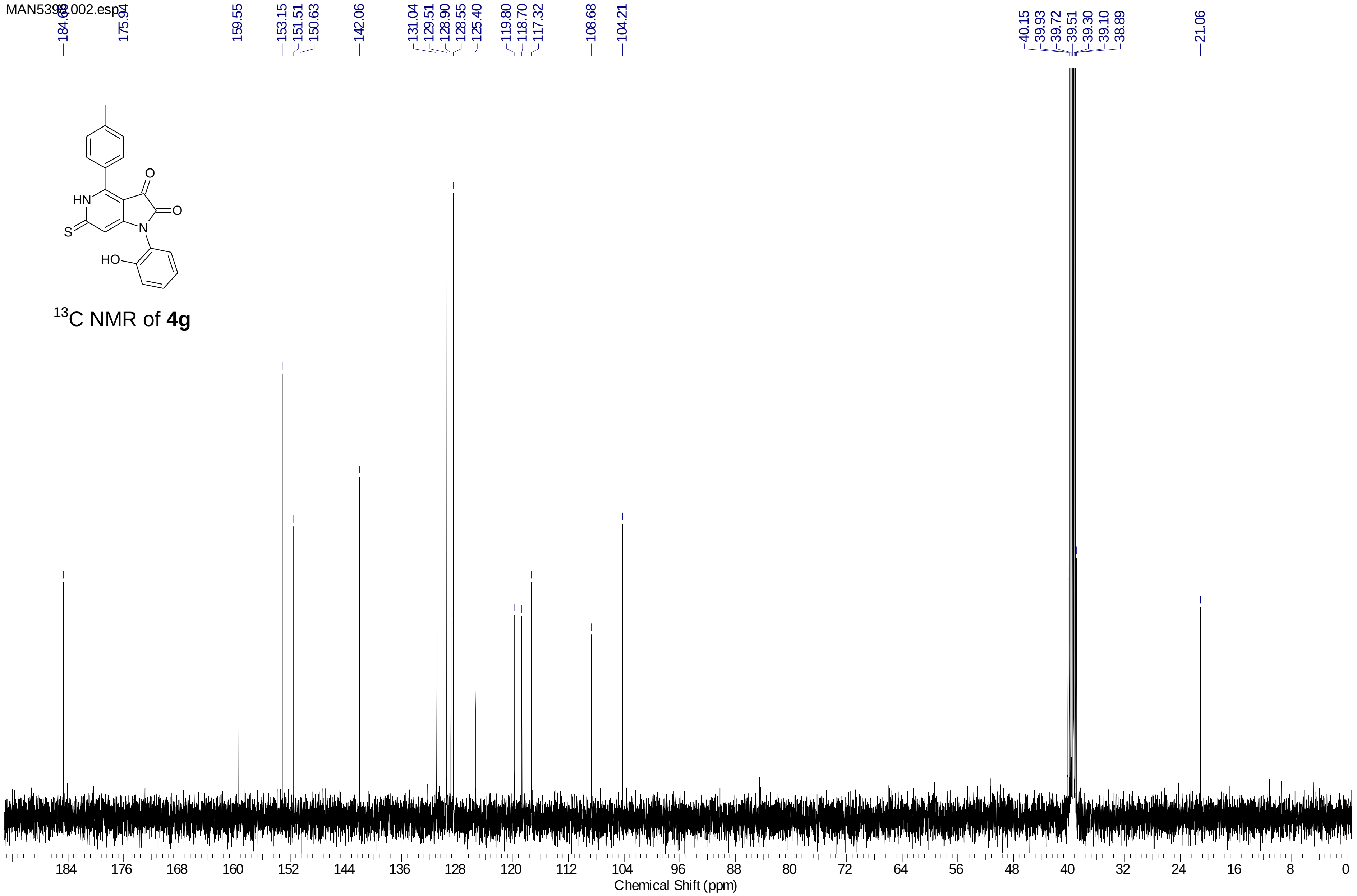
MAN5398.002.esp
184.68
175.94
159.55
153.15
151.51
150.63
142.06
131.04
129.51
128.90
128.55
125.40
119.80
118.70
117.32
108.68
104.21
40.15
39.93
39.72
39.51
39.30
39.10
38.89
21.06
184
176
168
160
152
144
136
128
120
112
104
96
88
80
72
64
56
48
40
32
24
16
8
0
Chemical Shift (ppm)
HN
O
O
N
S
HO

^{13}C NMR of **4g**

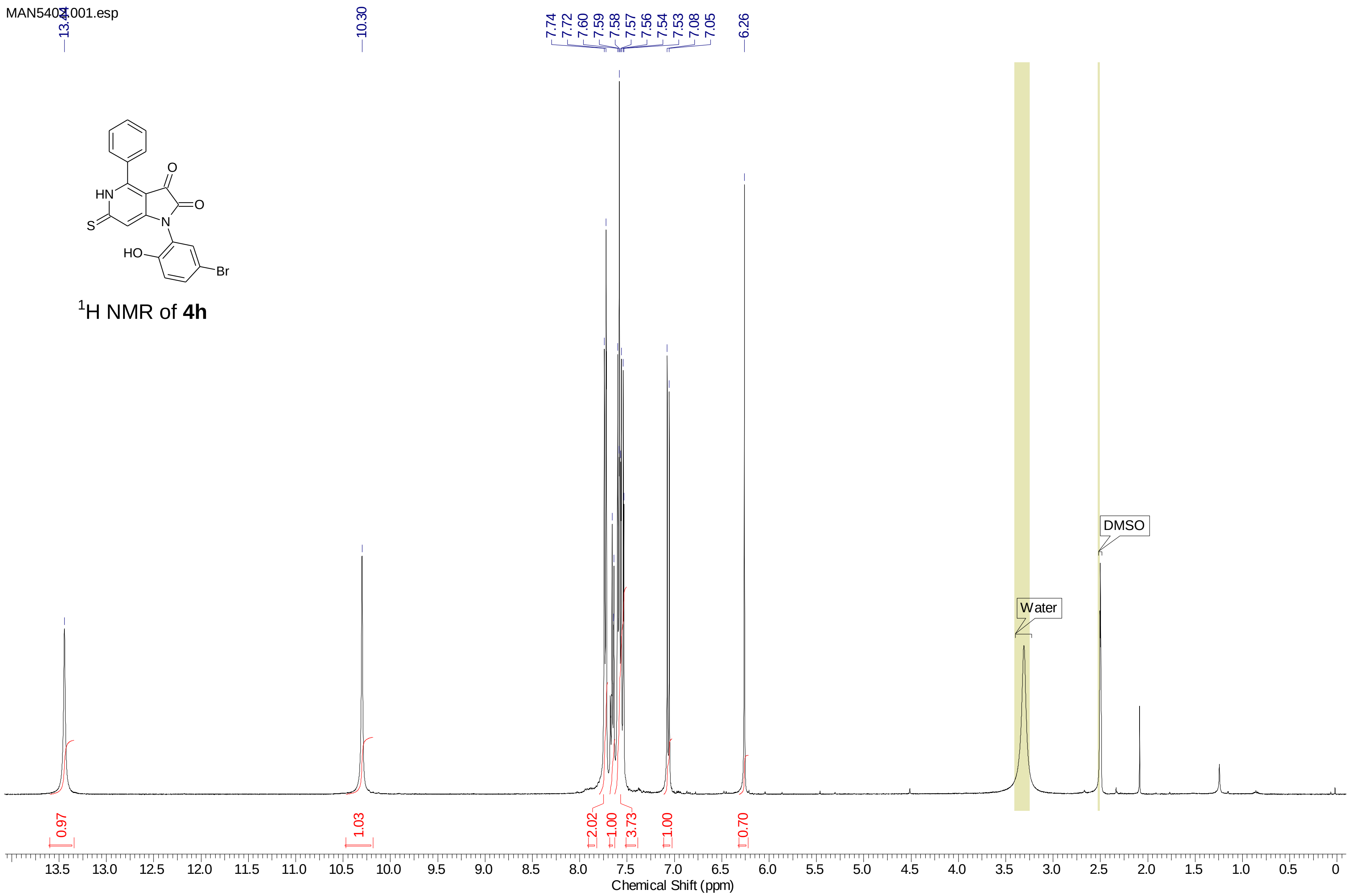
MAN5404001.esp
13.44
10.30
7.74
7.72
7.60
7.59
7.58
7.57
7.56
7.54
7.53
7.08
7.05
6.26
HN
O
O
S
N
HO
Br
Water
DMSO
0.97
1.03
2.02
1.00
3.73
1.00
0.70
13.5 13.0 12.5 12.0 11.5 11.0 10.5 10.0 9.5 9.0 8.5 8.0 7.5 7.0 6.5 6.0 5.5 5.0 4.5 4.0 3.5 3.0 2.5 2.0 1.5 1.0 0.5 0
Chemical Shift (ppm)

^{1}H NMR of **4h**

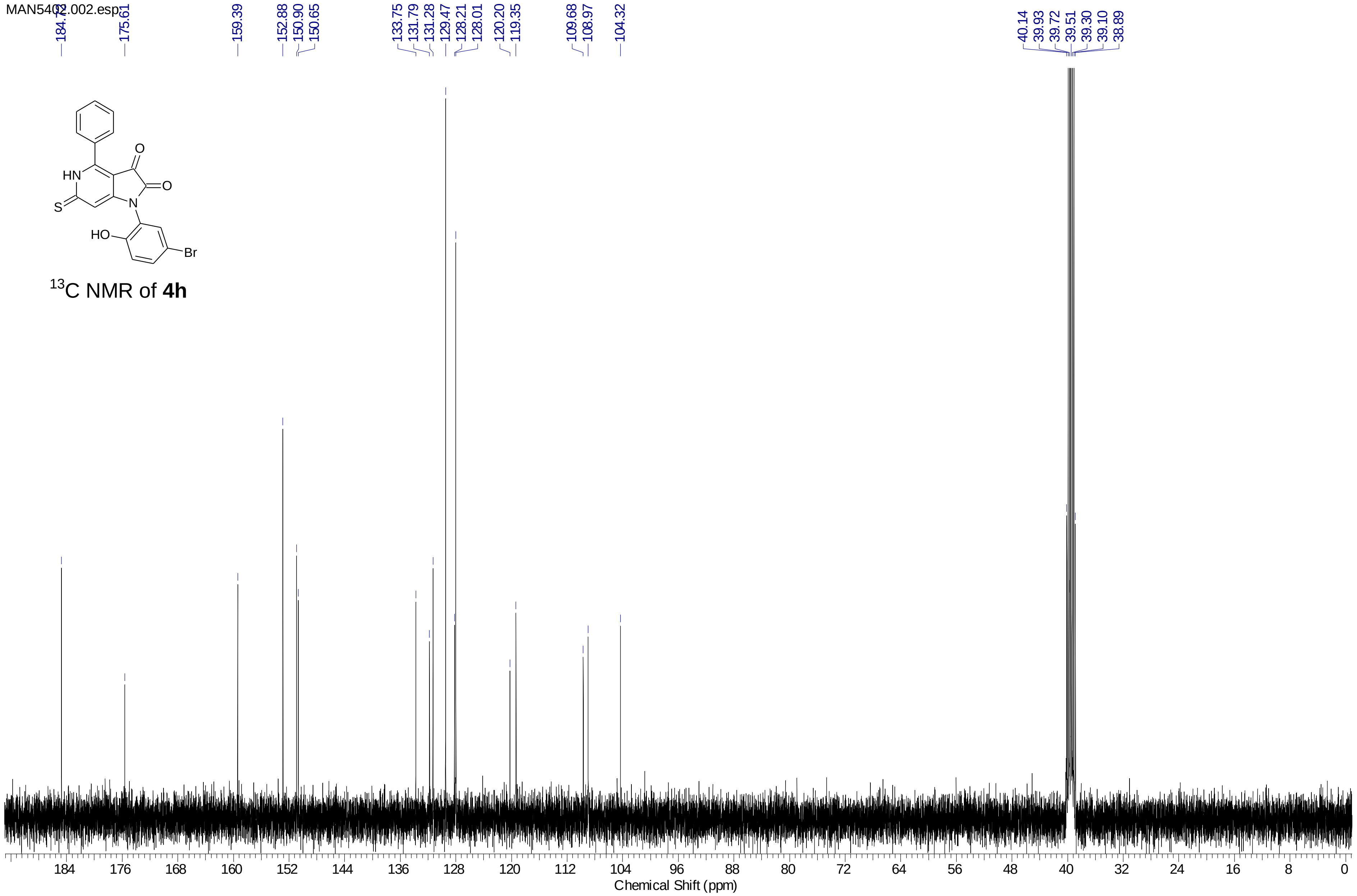
MAN5402.002.esp
184.72
175.61
159.39
152.88
150.90
150.65
133.75
131.79
131.28
129.47
128.21
128.01
120.20
119.35
109.68
108.97
104.32
40.14
39.93
39.72
39.51
39.30
39.10
38.89
HN
O
O
N
S
HO
Br
13C NMR of 4h
184
176
168
160
152
144
136
128
120
112
104
96
88
80
72
64
56
48
40
32
24
16
8
0
Chemical Shift (ppm)

MAN5416.001.esp

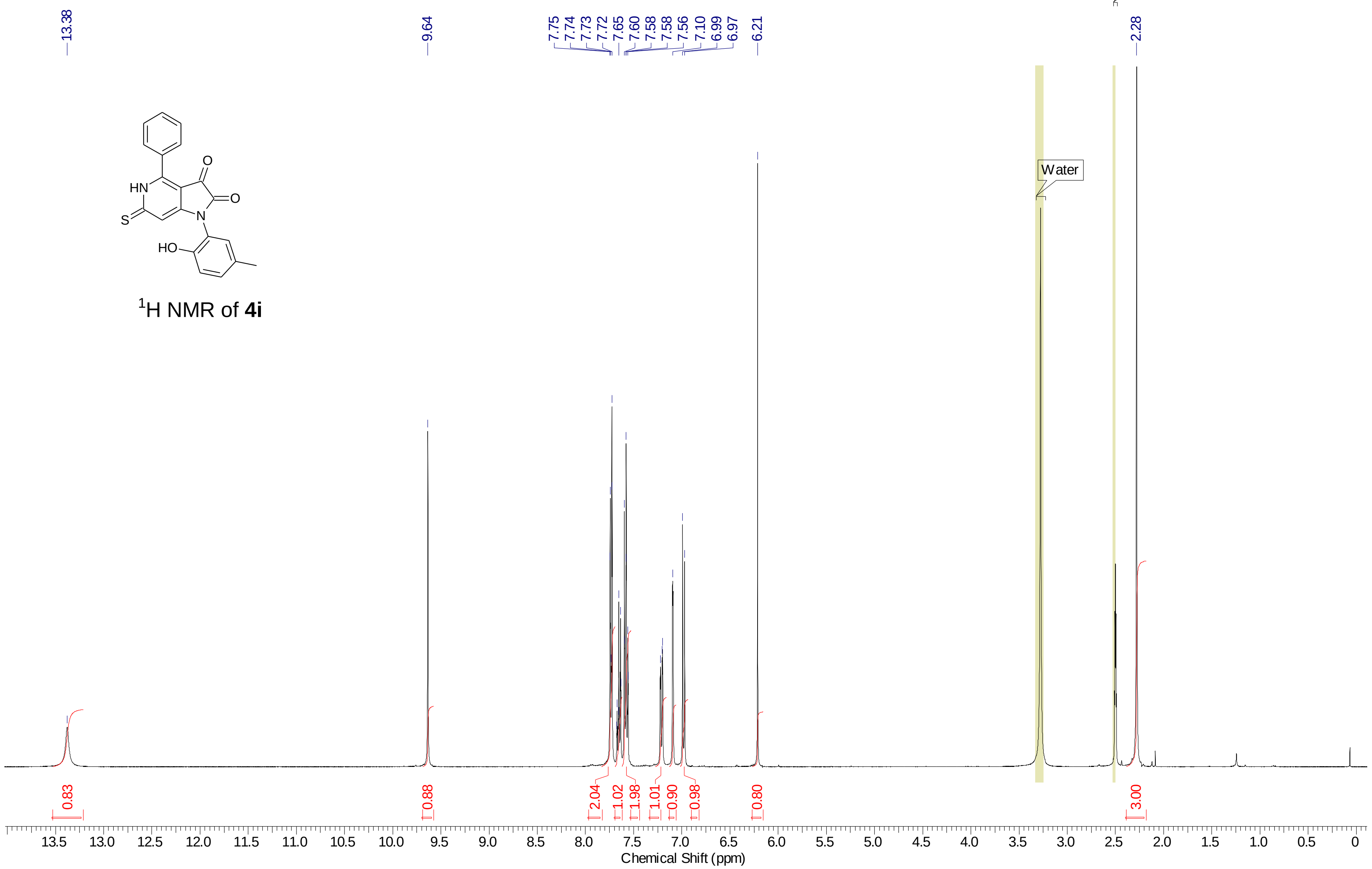

^{1}H NMR of **4i**

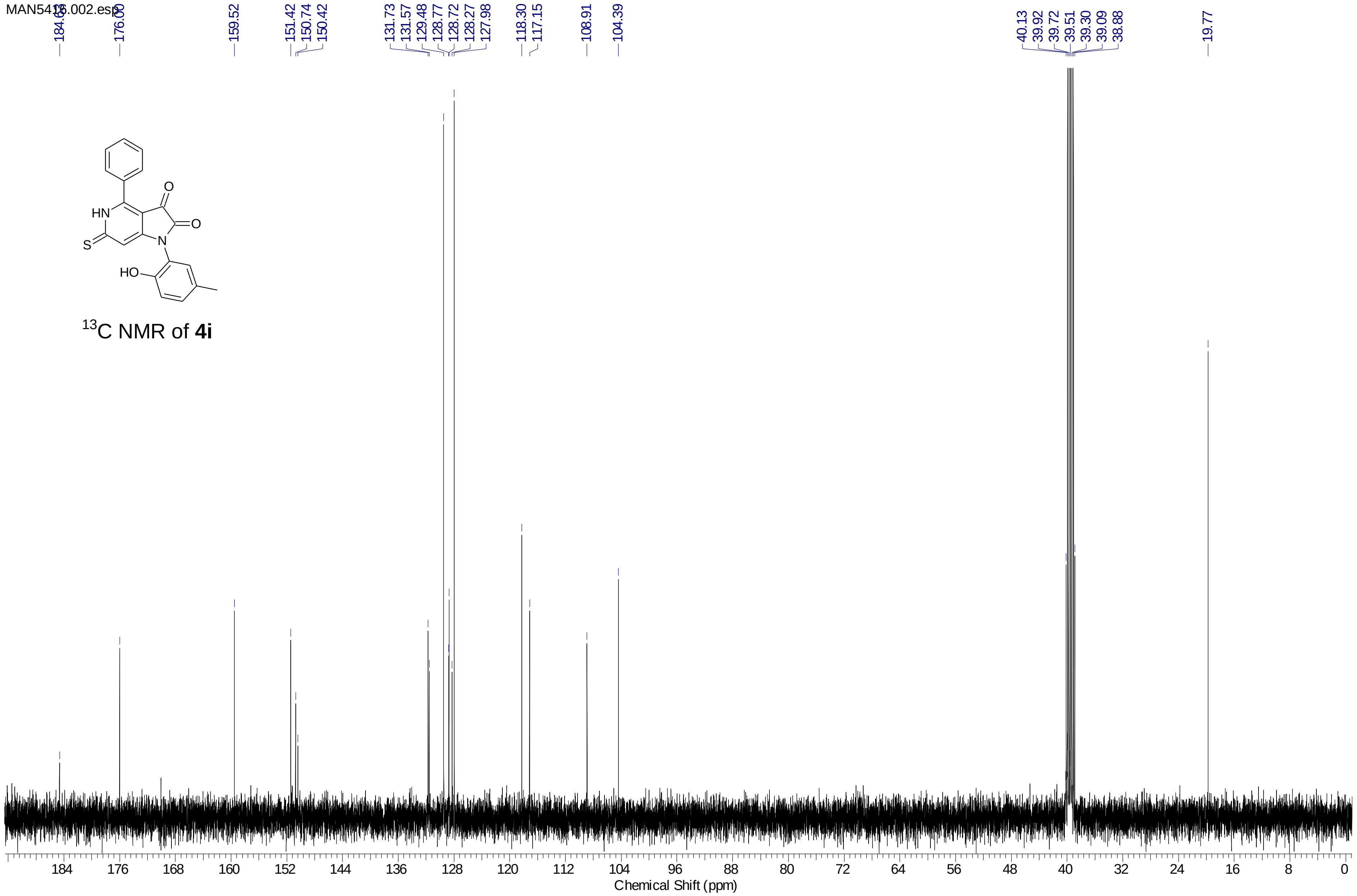
MAN5416.002.esp
184.65
176.00
159.52
151.42
150.74
150.42
131.73
131.57
129.48
128.77
128.72
128.27
127.98
118.30
117.15
108.91
104.39
40.13
39.92
39.72
39.51
39.30
39.09
38.88
19.77
HN
O
O
N
S
HO
^{13}C NMR of **4i**
184
176
168
160
152
144
136
128
120
112
104
96
88
80
72
64
56
48
40
32
24
16
8
0
Chemical Shift (ppm)

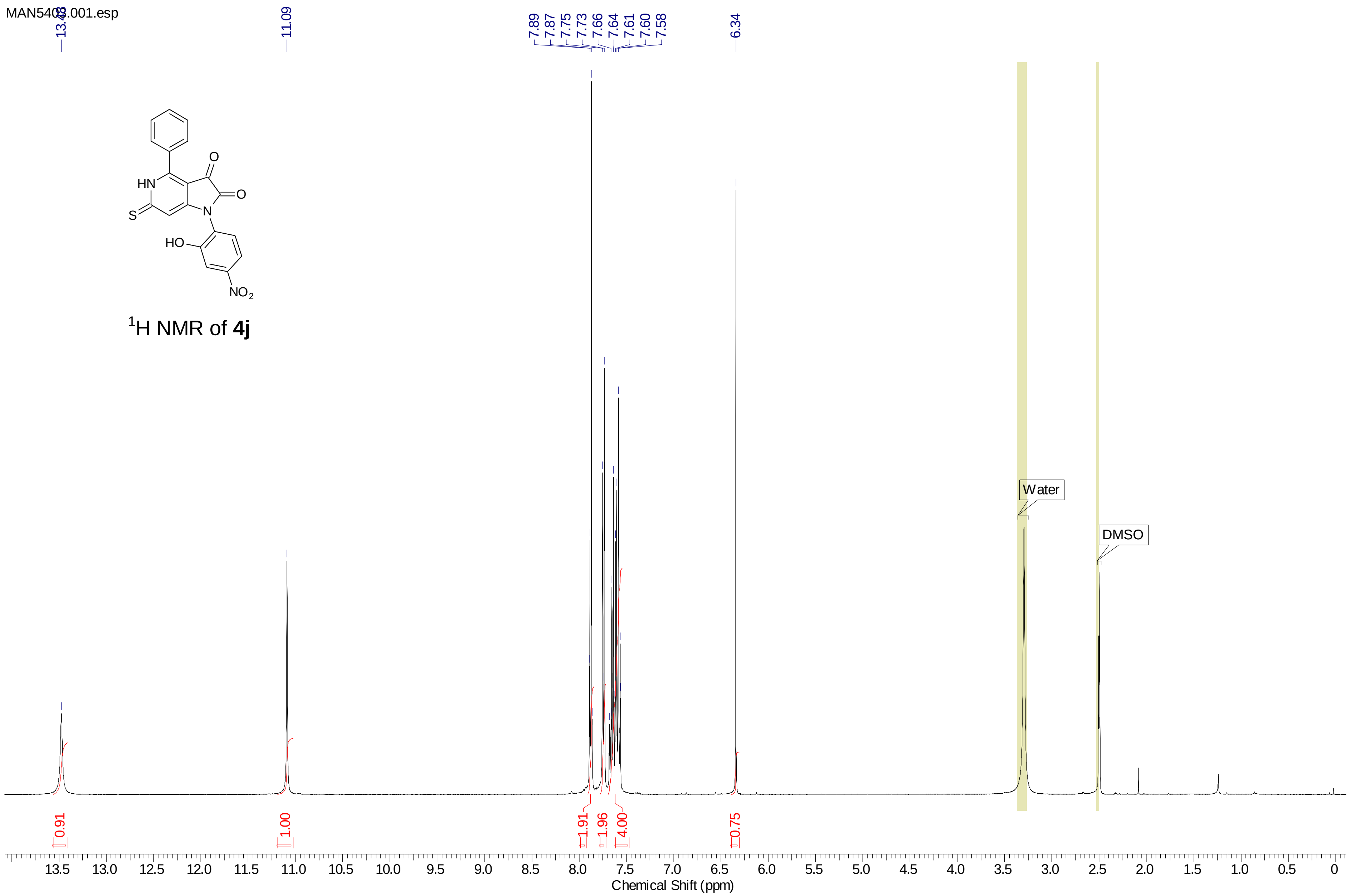

13.48
11.09
7.89
7.87
7.75
7.73
7.66
7.64
7.61
7.60
7.58
6.34
HN
O
O
N
S
HO
NO_2
^{1}H NMR of **4j**
Water
DMSO
0.91
1.00
1.91
1.96
4.00
0.75
13.5 13.0 12.5 12.0 11.5 11.0 10.5 10.0 9.5 9.0 8.5 8.0 7.5 7.0 6.5 6.0 5.5 5.0 4.5 4.0 3.5 3.0 2.5 2.0 1.5 1.0 0.5 0
Chemical Shift (ppm)

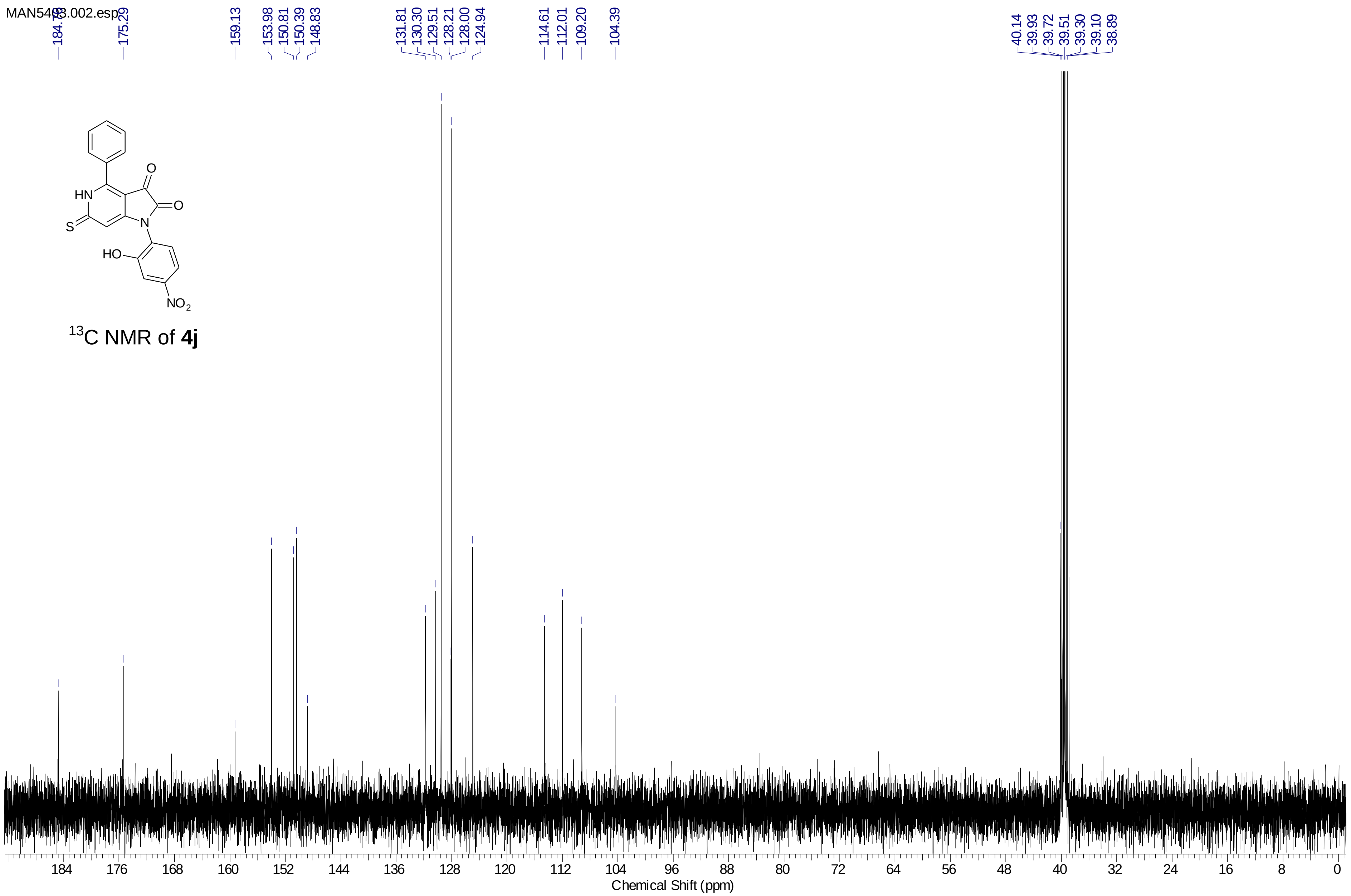
MAN5493.002.esp
184.78
175.29
159.13
153.98
150.81
150.39
148.83
131.81
130.30
129.51
128.21
128.00
124.94
114.61
112.01
109.20
104.39
40.14
39.93
39.72
39.51
39.30
39.10
38.89
HN
O
O
N
S
HO
NO2
184 176 168 160 152 144 136 128 120 112 104 96 88 80 72 64 56 48 40 32 24 16 8 0
Chemical Shift (ppm)

^{13}C NMR of **4j**

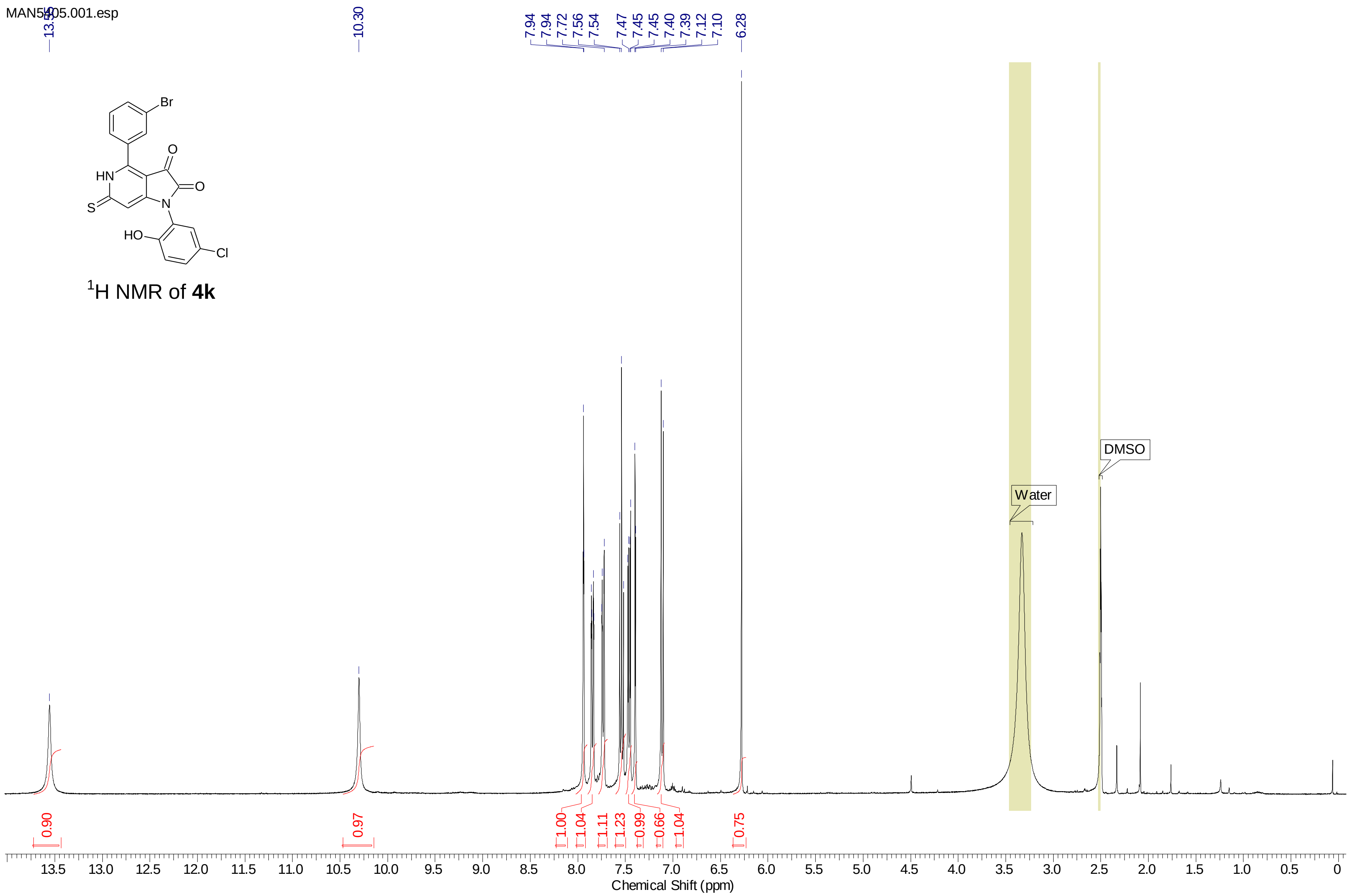
MAN5405.001.esp
13.54
10.30
7.94
7.94
7.72
7.56
7.54
7.47
7.45
7.45
7.40
7.39
7.12
7.10
6.28
Br
O
HN
O
N
S
HO
Cl
¹H NMR of 4k
Water
DMSO
0.90
0.97
1.00
1.04
1.11
1.23
0.99
0.66
1.04
0.75
13.5
13.0
12.5
12.0
11.5
11.0
10.5
10.0
9.5
9.0
8.5
8.0
7.5
7.0
6.5
6.0
5.5
5.0
4.5
4.0
3.5
3.0
2.5
2.0
1.5
1.0
0.5
0
Chemical Shift (ppm)

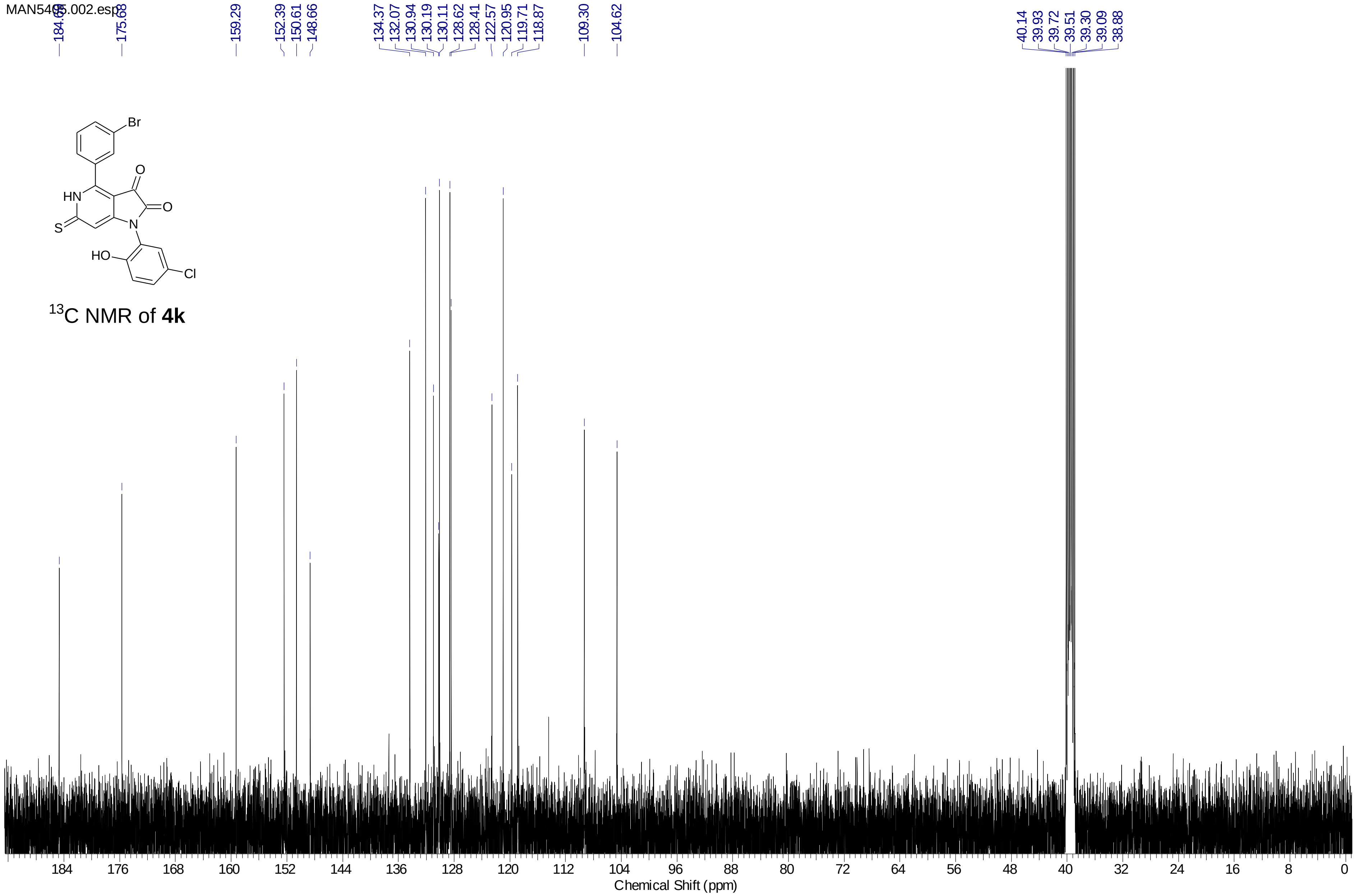
184.69
175.68
159.29
152.39
150.61
148.66
134.37
132.07
130.94
130.19
130.11
128.62
128.41
122.57
120.95
119.71
118.87
109.30
104.62
40.14
39.93
39.72
39.51
39.30
39.09
38.88
Br
O
HN
O
N
S
HO
Cl
13C NMR of 4k
184
176
168
160
152
144
136
128
120
112
104
96
88
80
72
64
56
48
40
32
24
16
8
0
Chemical Shift (ppm)

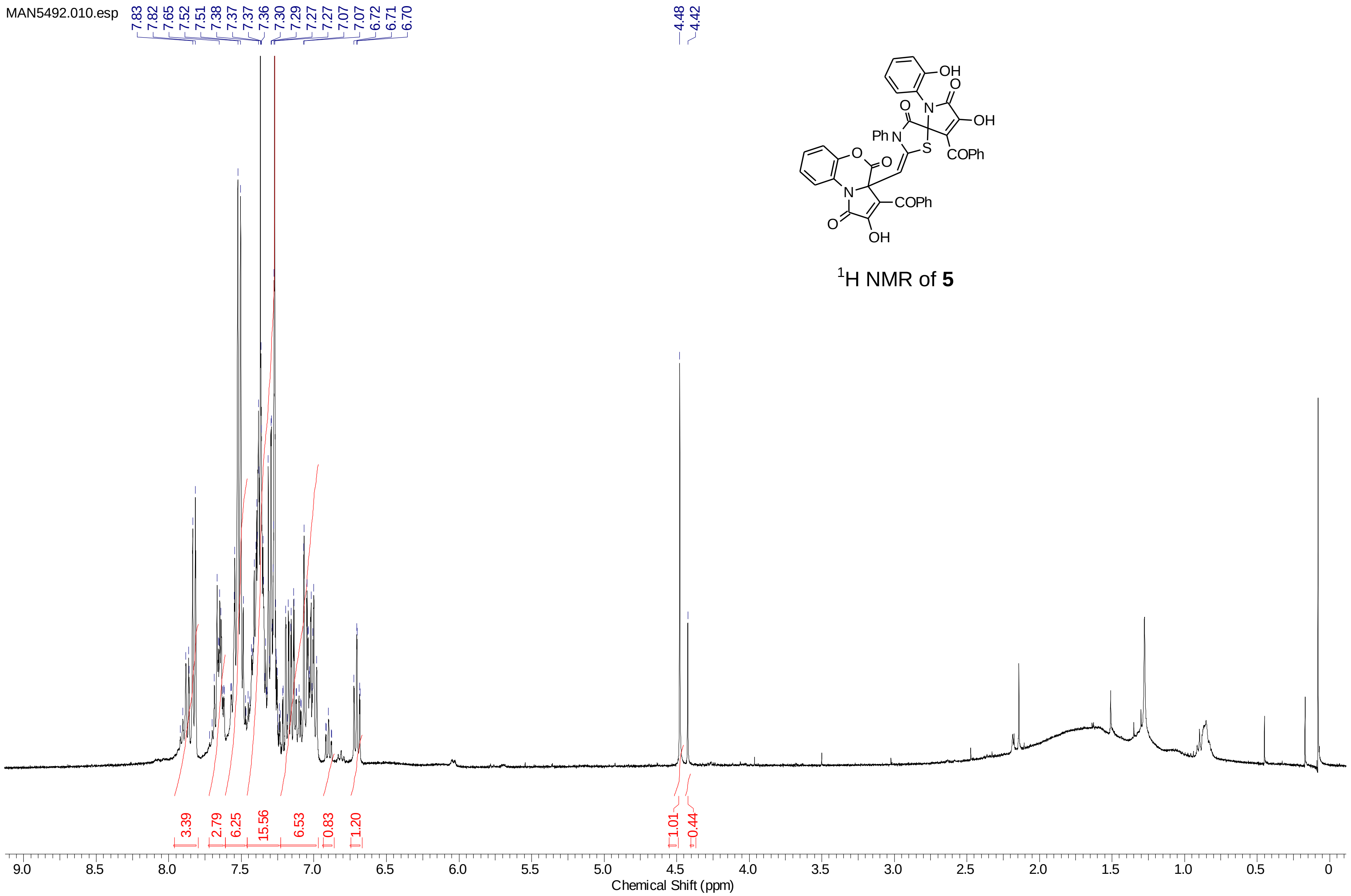
MAN5492.010.esp
7.83
7.82
7.65
7.52
7.51
7.38
7.37
7.37
7.36
7.30
7.29
7.27
7.27
7.07
7.07
6.72
6.71
6.70
4.48
4.42
OH
O
N
OH
O
Ph N
S
COPh
O
O
N
COPh
O
OH
3.39
2.79
6.25
15.56
6.53
0.83
1.20
1.01
0.44
9.0
8.5
8.0
7.5
7.0
6.5
6.0
5.5
5.0
4.5
4.0
3.5
3.0
2.5
2.0
1.5
1.0
0.5
0
Chemical Shift (ppm)

^{1}H NMR of **5**

Crystal structure determination

The unit cell parameters and the X-ray diffraction intensities were measured on a Xcalibur Ruby diffractometer. The empirical absorption correction was introduced by multi-scan method using SCALE3 ABSPACK algorithm[2]. Using OLEX2[3], the structures were solved with the SHELXT program[4] (for **4c**) or with the Superflip program[5] (for **5**) and refined by the full-matrix least-squares method in the anisotropic approximation for all non-hydrogen atoms using the SHELXL program[6]. The hydrogen atoms were treated by a mixture of independent and constrained refinement. The contribution of the solvent electron density (for **5**) was removed (except for one molecule of acetone) using the SQUEEZE routine in PLATON[7].

Crystal Data of **4c**. $C_{21}H_{16}N_2O_4S$, M = 392.42, orthorhombic, a = 33.376(9) Å, b = 17.309(5) Å, c = 6.5958(14) Å, V = 3810.5(17) Å^3, T = 295(2), space group Pccn, Z = 8, μ(Mo Kα) = 0.200 mm^{-1}. The final refinement parameters: R_1 = 0.0892, wR_2 = 0.2006 [for observed 1759 reflections with $I > 2\sigma(I)$]; R_1 = 0.1918, wR_2 = 0.2788 (for all independent 4567 reflections, R_{int} = 0.1686), S = 0.963.

Crystal Data of **5**. $C_{44}H_{27}N_3O_{10}S \cdot C_3H_6O$ (without solvent), M = 847.82, triclinic, a = 11.4101(11) Å, b = 15.3624(16) Å, c = 18.0952(16) Å, α = 69.836(9)°, β = 82.906(7)°, γ = 68.317(9)°, V = 2766.7(5) Å^3, T = 295(2), space group P–1, Z = 2, μ(Mo Kα) = 0.109 mm^{-1}. The final refinement parameters: R_1 = 0.0581, wR_2 = 0.1477 [for observed 7923 reflections with $I > 2\sigma(I)$]; R_1 = 0.0969, wR_2 = 0.1682 (for all independent 13026 reflections, R_{int} = 0.0304), S = 1.061.

CCDC 1877232 (**4c**) and 1879686 (**5**) contain the supplementary crystallographic data for this paper. The data can be obtained free of charge from The Cambridge Crystallographic Data Centre via http://www.ccdc.cam.ac.uk.

2. CrysAlisPro, Agilent Technologies, Version 1.171.37.33 (release 27-03-2014 CrysAlis171 .NET).
3. Dolomanov, O.V., Bourhis, L.J., Gildea, R.J, Howard, J.A.K., Puschmann, H. *J. Appl. Cryst.* **2009**, *42*, 339.
4. Sheldrick G.M. *ActaCryst*. **2015**, *A71*, 3.
5. Palatinus L., Chapuis G. *J. Appl. Cryst.* **2007**, *40*, 786.
6. Sheldrick, G.M. *ActaCryst*. **2015**, *C71*, 3.
7. Spek A. L., *Acta Cryst.* **2015**, *C71*, 9.
